# Projective light-sheet microscopy with flexible parameter selection

**DOI:** 10.1038/s41467-024-46693-y

**Published:** 2024-03-29

**Authors:** Bingying Chen, Bo-Jui Chang, Stephan Daetwyler, Felix Zhou, Shiv Sharma, Donghoon M. Lee, Amruta Nayak, Jungsik Noh, Konstantin Dubrovinski, Elizabeth H. Chen, Michael Glotzer, Reto Fiolka

**Affiliations:** 1 Lyda Hill Department of Bioinformatics, University of Texas Southwestern Medical Center, Dallas, TX USA; 2 Department of Molecular Biology, University of Texas Southwestern Medical Center, Dallas, TX USA; 3 Department of Molecular Genetics and Cell Biology, University of Chicago, Chicago, IL USA; 4 Department of Biophysics, University of Texas Southwestern Medical Center, Dallas, TX USA; 5 Department of Cell Biology, University of Texas Southwestern Medical Center, Dallas, TX USA; 6 Hamon Center for Regenerative Science and Medicine, University of Texas Southwestern Medical Center, Dallas, TX USA

**Keywords:** Fluorescence imaging, Optical imaging, Neurological models

## Abstract

Projection imaging accelerates volumetric interrogation in fluorescence microscopy, but for multi-cellular samples, the resulting images may lack contrast, as many structures and haze are summed up. Here, we demonstrate rapid projective light-sheet imaging with parameter selection (props) of imaging depth, position and viewing angle. This allows us to selectively image different sub-volumes of a sample, rapidly switch between them and exclude background fluorescence. Here we demonstrate the power of props by functional imaging within distinct regions of the zebrafish brain, monitoring calcium firing inside muscle cells of moving Drosophila larvae, super-resolution imaging of selected cell layers, and by optically unwrapping the curved surface of a Drosophila embryo. We anticipate that props will accelerate volumetric interrogation, ranging from subcellular to mesoscopic scales.

## Abstract (web summary)

Projection imaging for multi-cellular samples can be hindered by several factors, including low contrast. Here, the authors propose projective light-sheet imaging with parameter selection (props) of imaging depth, position and viewing angle.

## Introduction

Fluorescence microscopy allows observations of cellular and subcellular dynamics with high specificity and sensitivity in 3D. Traditionally, acquiring volumetric data involves recording a series of 2D images while stepping the focal plane through the sample or mechanically moving the sample through a stationary focal plane. Consequently, this process can be too slow for rapid dynamics, and its sequential nature prevents simultaneous observations across an entire volume. As an alternative, forming a rapid 2D projection of a 3D volume can increase the volumetric interrogation rate and ensure simultaneity of observation.

Typically, a projection technique extends the depth of focus of an imaging system, either through PSF engineering or by dynamically sweeping the focal plane to capture the whole volume within one image exposure. If PSF engineering is performed with a phase mask^1^, the depth of focus extension is fixed. Focal sweeping has been performed with electro-tunable lenses and tunable acoustic gradient index of refraction (TAG) lenses^2^. However, such devices are singlet lenses that can only introduce a quadratic phase function, which in turn can lead to spherical and field-dependent aberrations^3^. Remote focusing techniques that can compensate for such aberrations have been developed^4^, but fluorescence light losses occur due to the use of polarizing beam splitters. Further, any fluorescence stemming from outside the depth of focus or from scattered light cannot be suppressed, except for multi-photon excitation methods^5^. Nevertheless, multi-photon excitation comes with its own caveats, such as high laser powers, complex and expensive light sources, and a limited choice of fluorophores.

As another option, we have recently introduced a multi-angle projection technique that can provide high image contrast^6^ when implemented in a light-sheet microscope^7^ as the sample is optically sectioned by the light-sheet. However, for multi-cellular samples, light scattering can lead to background haze. Therefore, even light-sheet based projection imaging has been limited to rather sparse and transparent samples, as otherwise, the image quality degrades rapidly.

Here, we introduce a projection imaging method where the depth of focus and axial position can be adjusted on the fly, and background fluorescence is robustly rejected. More specifically, a selected volume of variable depth and location within the sample can be projected on a camera, and fluorescence from outside this volume is suppressed, leading to sharper images than would result from a projection of the whole sample. Further, when combined with oblique plane microscopy (OPM)^8^, projections at different z-positions can be rapidly acquired without the movement of the sample or objective. We call the method projections with parameter selection, or props for short, which is added as a suffix to the microscope technique being used, e.g., OPMprops. We show how props is applicable from subcellular imaging of single cells to mesoscopic imaging of whole organisms and highlight its rapid imaging capability by recording neuronal firing across different brain layers and muscle contractions inside moving organisms.

## Results

### Principle of projection imaging with parameter selection (props)

**Fig. 1:**
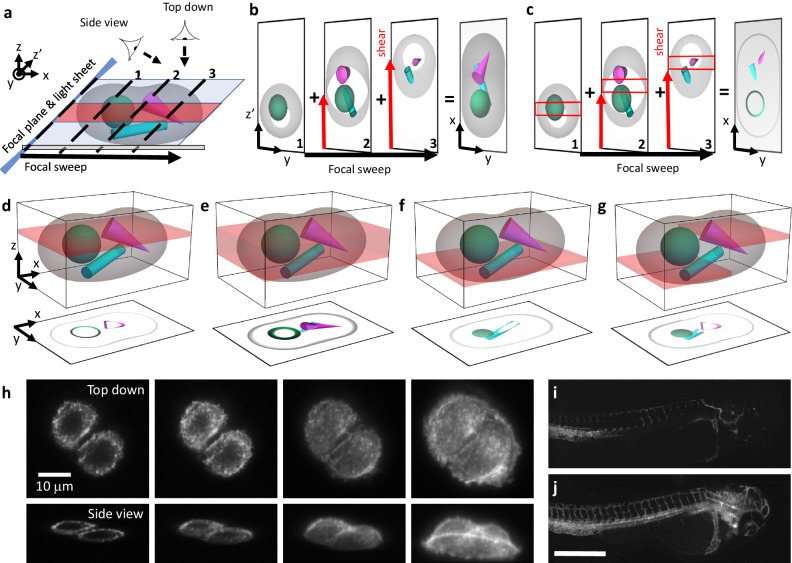
Concept of projections with parameter selection (props). **a** Schematic overview of the imaging geometry in oblique plane microscopy (OPM) and lattice light-sheet microscopy (LLSM). The sample is rapidly scanned with a light sheet (blue) and corresponding focal plane, which is termed a “focal sweep”. If the focal sweep is completed during one camera exposure, an optical projection is formed. **b** Translating the image on the camera (red arrow labeled “shear”) during a focal sweep results in projections under different viewing angles. On the right, a top-down projection along the *z*-axis is shown. **c** When a rolling shutter (red boxes) is synchronized to the shear translation along the camera, a projection of a sub-volume (red shaded area in **a**) is formed. **d**, **e** Schematic illustration of varying the projection depth with the rolling shutter width. The red-shaded volumes illustrate the regions of the sample that are projected below, illustrated as a top-down view. **f** Illustration of shifting the projection volume axially by introducing a time delay between the rolling shutter and the focal sweep and shearing. **g** A nonlinear scanning waveform results in an axial change within the projection volume. **h** LLSMprops imaging of A375 cells labeled with F-tractin-EGFP with varying projection depth, top row: top-down view, bottom row: corresponding 45° viewing angle. The imaging experiment was repeated independently 3 times with similar results. **i**, **j** Mesoscopic OPMprops imaging of zebrafish vasculature, labeled with Tg(kdrl:EGFP), over a projection depth of 4 and 362 microns, respectively. The imaging experiment was repeated independently 4 times with similar results. Scale Bar: **h**: 10 microns, **j**: 500 microns.

Projection imaging with parameter selection (props, schematic in Fig. 1) builds on our previously introduced rapid projection imaging method^6^, which can be advantageously combined with lattice light sheet microscopy (LLSM) or oblique plane microscopy (OPM)^8,9^. The sample volume is quickly scanned with a tilted light-sheet and the corresponding focal plane, which we refer to as a focal sweep (Fig. 1a). Of note, this is a geometry representative for both OPM and LLSM, i.e., the scan direction is along the coverslip (here denoted as the *x*-direction), but the light-sheet and focal plane are tilted to the scan axis.

A projection of the volume is formed when the focal sweep occurs during one camera exposure. As we have shown previously, by scanning the fluorescence image over the camera chip during the focal sweep (Fig. 1b, see also Supplementary Fig. 1a), the viewing angle of such a projection can be adjusted^6^, leveraging the shear warp transform^10^. In the illustration shown in Fig. 1b, the viewing angle of the resulting projection is along the z-axis, referred to in this manuscript as a top-down view.

However, such projections contain the entirety of the sample (blue shaded volume in Fig. 1a), and anything beyond that may get excited, leading to blurry images in complex, multi-cellular samples. Further, for feature-rich samples, a projection over the whole sample volume may lead to too many overlapping features, obscuring local details. To address this issue, we applied a rolling shutter readout to the camera during the scanning over the camera chip (the “shear” in the shear warp transform, shown as a red arrow in Fig. 1b, c)^11–13^. As such, we can form projections from a sub-volume of the sample (red shaded slab in Fig. 1a; Supplementary Fig. 1b, c). The extent of the sub-volume, as well as its axial position (i.e., in the *z*-direction), can be freely chosen based on the camera and scan parameters. Increasing the rolling shutter width yields projections over a larger depth (Fig. 1d, e; Supplementary Fig. 2 and Supplementary Movies 1–3). When a time delay is introduced between the rolling shutter and the focal sweep, the axial position of the projection is varied (Fig. 1f; Supplementary Figs. 1, 3, and 4). Lastly, if the shear galvo is driven by a nonlinear waveform, the axial position of the projection can be changed during a single scan. This enables adaptive projection imaging to fit the projected volume to the sample geometry (Fig. 1g; Supplementary Notes 1 and 2 and Supplementary Figs. 3 and 5–8).

### Props allows adjustable projection depth

To demonstrate the ability of props to change the projection depth, we implemented it on two microscopes: a lattice light-sheet microscope (LLSMprops) and a mesoscopic oblique plane microscope that can capture whole organisms (meso OPMprops)^14^. Using LLSMprops, we imaged two A375 cells labeled with F-tractin-EGFP (Fig. 1h). Props allow dynamic changes to the projection depth, ranging from a confocal slice of the two A375 cells up to their entirety. While a top-down view (i.e., along the *z*-axis, top row in Fig. 1h) cannot easily reveal the depth range we can cover, we exploited the fact that we can also change the viewing angle in our method. Under a 45° viewing angle, changes in depth range became more evident (Fig. 1h, bottom row).

Importantly, the depth of the projection is decoupled from the thickness of the light sheet or the depth of focus of the detection system. Instead, it is limited by the volume the light sheet can cover (see Fig. 1a, i.e., the height above the coverslip) and the transparency of the sample. This allowed us to vary the projection depth in zebrafish larvae with the vascular marker, Tg(kdrl:EGFP), from 4 to 362 microns using meso OPMprops (Fig. 1i, j, Supplementary Movies 1 and 2). The depth coverage thus can exceed conventional imaging modalities (confocal and light-sheet microscopes typically have 0.5–2 microns depth of focus) by two orders of magnitude.

**Fig. 2:**
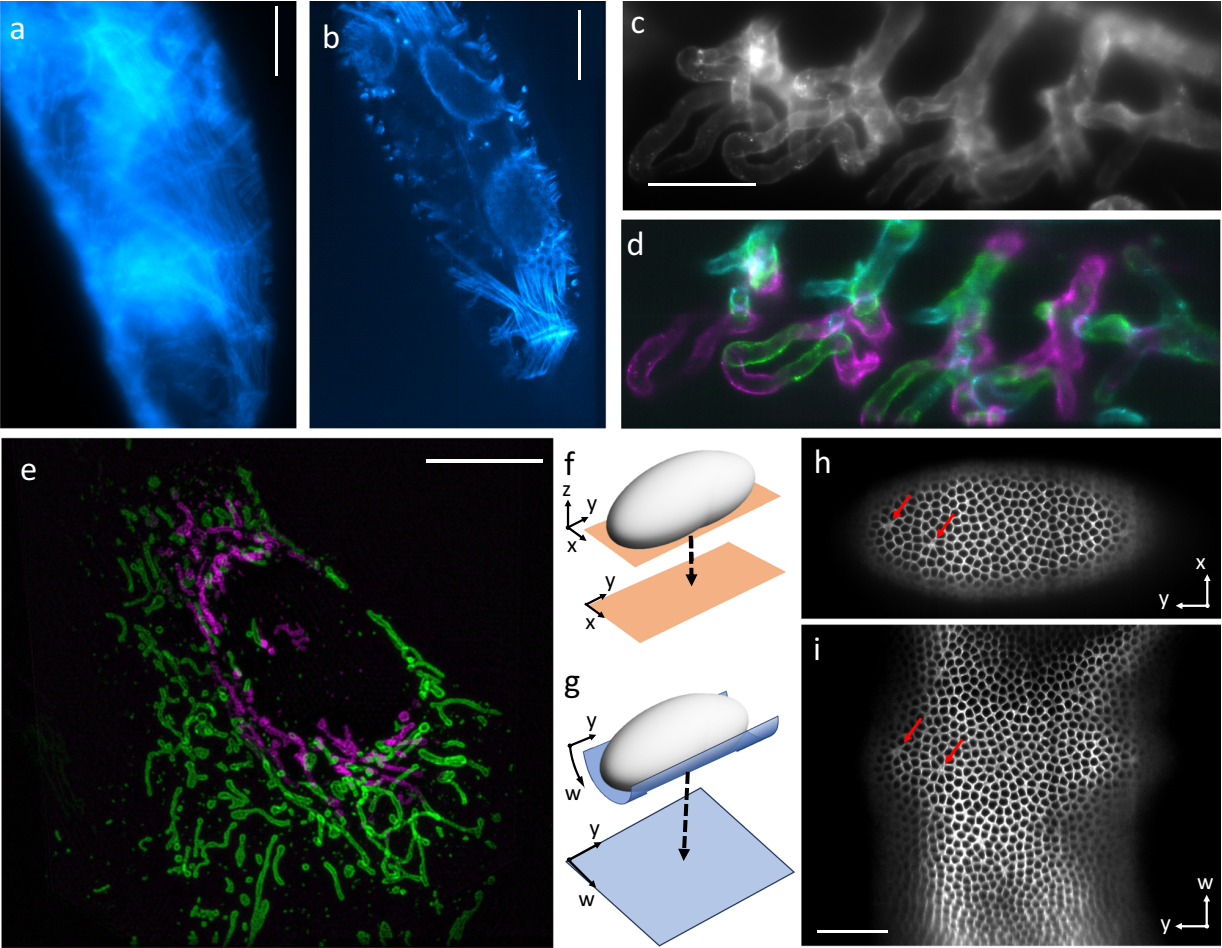
Variation of projection parameters on different biological samples. **a** Projection of a Drosophila embryo labeled with UAS-actin5C-RFP using OPMpro. **b** OPMprops projection of the same embryo. **c** Projection of the gill vasculature labeled with the vascular marker Tg(*kdrl:Hsa.HRAS-mCherry*) in a zebrafish larva using OPMpro. **d** Three sequentially acquired projections using OPMprops. The projections divide the volume in **c** into sub-compartments, which are color-coded in magenta, green, and cyan, respectively. Both the Drosophila and zebrafish imaging were repeated independently 5 times with similar results. **e** A single osteosarcoma (U-2 OS) cell, as imaged with oblique plane structured illumination microscopy (OPSIM) using props. Green are mitochondria in a projection layer adjacent to the coverslip, magenta are mitochondria in a projection layer 4 microns above the coverslip. The two projections were acquired sequentially. The imaging experiment was repeated independently 3 times with similar results. **f** Schematic representation of imaging an *x*–*y* layer (orange) near the bottom of a Drosophila embryo (gray) that is mapped in a top-down view (dotted arrow). **g** Schematic representation of a curved surface (blue) that is mapped into a planar projection (dotted arrow). **h** XY projection of a Drosophila embryo labeled with myosin-FRB-GFP. **i** Curved projection of the same embryo. Red arrows point to selected features visible in both (**h**) and (**i**). The imaging experiment was repeated independently 4 times with similar results. Scale Bars: **a**–**c**: 50 microns, **e**: 10 microns, **i**: 50 microns.

The ability to vary the projection depth also allows the suppression of image blur in scattering samples. When imaging a conventional projection image of a *Drosophila* embryo expressing Actin5C-RFP, the background haze occluded many fine details due to light-scattering (Fig. 2a). The blur occurs as the projection image integrates fluorescence even beyond a depth in the embryo that can be accessed with a light-sheet microscope. In contrast, an OPMprops image (Fig. 2b) is strikingly clearer, as much of the background fluorescence is excluded.

### Props enables projection imaging with an axial offset

Props provides the ability to vary the axial position from where a projection is formed (Fig. 1f). Using OPMpro, we acquired a projection image of the gill vasculature in a zebrafish larva 3 days post fertilization (dpf), labeled with *Tg(kdrl:Hsa.HRAS-mCherry)* (Fig. 2c). By phase shifting the galvo signals with respect to the rolling shutter, we obtained three sequentially acquired projections of sub-volumes using OPMprops (Fig. 2d). These three sub-volumes partition the overall volume that was captured in Fig. 2c. This ability can also be applied to subcellular imaging, which we demonstrate on oblique plane structured illumination microscopy (OPSIM)^15^ in a projection format^16^ (Fig. 2e and Supplementary Fig. 4). With OPSIMprops, we acquired projections of two sub-volumes of a U-2 OS osteosarcoma cell labeled with OMP-GFP, an outer membrane marker for mitochondria. Thereby, we could separate mitochondria within a 2.5 microns thick layer adjacent to the coverslip (Fig. 2e, green) from mitochondria in a layer 4 microns above the coverslip (Fig. 2e, magenta). Importantly, previous forms of rapid 2D structured illumination, such as Total internal reflection fluorescence (TIRF) and Grazing incidence (GI) SIM, are locked to the coverslip surface and have a fixed imaging depth (100 nm and 1 micron for TIRF and GI modes, respectively)^17,18^. Here, using props, the region that is covered by a 2D SIM acquisition can be freely chosen and varied without the mechanical motion of the sample or objective.

### Sample geometry-driven props

By using non-linear waveforms to drive the galvo mirrors, projections along curved surfaces can be formed (Fig. 2g, Supplementary Note 2 and Supplementary Figs. 5–8). We leveraged this capability to optically unwrap the curved surface of a Drosophila embryo labeled with myosin-FRB-GFP (tagged at the endogenous locus) (Fig. 2f–i). In contrast to a conventional lateral projection (schematic: Fig. 2f, data: Fig. 2h), a curved projection (schematic: Fig. 2g, data: Fig. 2i and Supplementary Movie 4) captures a much larger portion of the embryo surface. Importantly, fitting a single projection of the sample to the geometry reduces the imaging time required compared to sequential imaging of several layers to capture a curved geometry. Supplementary Fig. 9 shows confocal images of the embryo acquired with a commercial spinning disk confocal microscope, where only some portions of its outer membrane can be captured in a single image acquisition. Projective imaging has been suggested as a way to compress data in embryo imaging^19^, but the projections were still formed numerically from 3D data streamed to a computer. Here, we show an optical way to directly form projections of curved surfaces at much higher rates.

### Rapid functional imaging with props

**Fig. 3:**
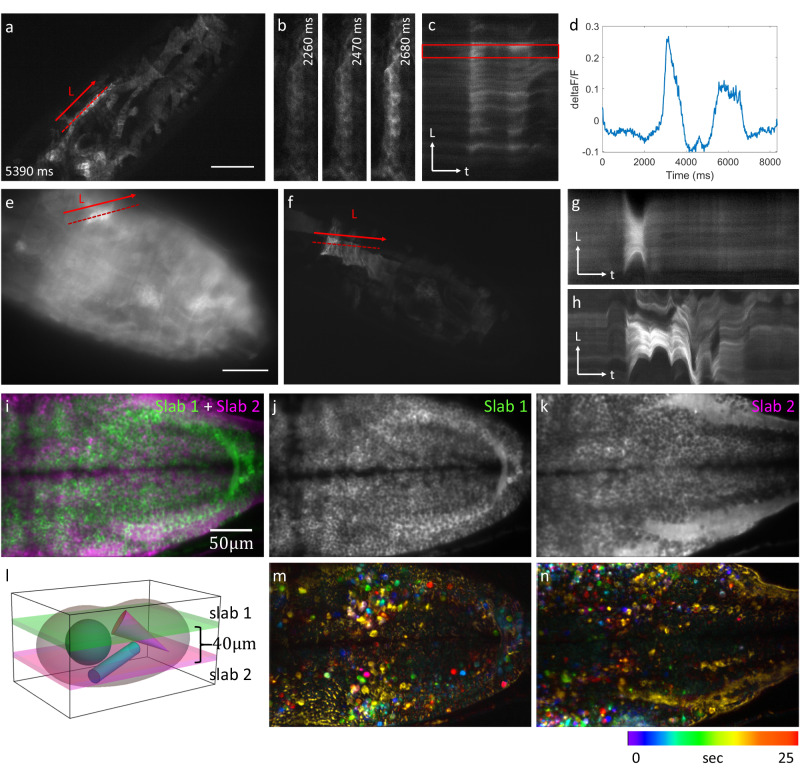
Selective projection imaging of calcium dynamics. **a**–**d** Calcium dynamics in a Drosophila embryo labeled with jGCaMP7s-CAAX imaged at a rate of 50 Hz using OPMprops. **b** Zoomed-in views of the area surrounding the dashed line in (**a**). **c** Kymograph along the dotted line in (**a**). **d** Calcium signal analysis (d*F*/*F*) from the box in (**c**). **e** Projection image of another jGCaMP7s-CAAX labeled embryo using OPMpro. **f** Same embryo as in (**e**), but imaged with OPMprops at 20 Hz framerate. **g** Kymograph along the dotted line in (**e**). **h** Kymograph along the dotted line in (**f**). **i**–**n** OPMprops imaging at two depths within a zebrafish larva labeled with Tg(elavl3:soma-GCaMP7f). **i** Overlay of the two projections. **j**, **k** Individual projections. **l** Schematic representation of the projection imaging. The two sub-volumes (“slabs”) are separated by 40 microns. **m**–**n** Color-coded timeseries corresponding to **j**, **k** where the static background was subtracted. Both the Drosophila embryo and zebrafish larva imaging were repeated independently 3 times with similar results. Source data are provided as a Source Data file.

Props can be used to follow dynamics that are too rapid to be captured by 3D stacking and in samples that are too optically dense for conventional projection imaging. To demonstrate fast projection imaging, we imaged calcium dynamics with OPMprops in *Drosophila* embryo expressing UAST-jGCaMP7s-CAAX at 50 Hz frame rate (Fig. 3a–d, Supplementary Movie 5), revealing rapid calcium firing events. We also observed muscle contractions and corresponding calcium signal increases over 1200 frames with a 20 Hz frame rate in a moving organism (Fig. 3e–h, see also Supplementary Movies 6 and 7). In contrast to conventional projection imaging (Fig. 3e, g), OPMprops revealed non-uniform distributions of calcium activity in the muscle cells more clearly (Fig. 3f, h).

For functional imaging of neuronal calcium signaling, OPMprops allowed us to rapidly image two layers of the zebrafish brain, separated in the axial direction by 40 microns (Fig. 3i–l, Supplementary Movie 8). The neurons were labeled with a somatic calcium sensor, Tg(*elavl3:soma-GCaMP7f*)^20^. The projection depth was adjusted to balance the number of neurons imaged at a time and the signal-to-background ratio (Supplementary Note 3 and Supplementary Figs. 10 and 11). Props allowed more imaging flexibility than a confocal or spinning disk microscope whose depth of focus is too shallow to contain the entire cell body of a neuron.

The switching between the two imaging layers happened within milliseconds, as no mechanical motion of the sample or objective was involved, enabling near-simultaneous observations of neuronal firing across different brain layers. Subsequent image processing with background subtraction enabled visualization of calcium signaling in individual neurons in both brain regions (Fig. 3m, n).

## Discussion

In summary, we have introduced projection imaging with parameter selection (props), which enables rapid projection imaging with adjustable depth and axial position. Thus, projections can cover selected sub-volumes of the sample, which can range from a projection of a thin confocal slice to the entire imaging volume. This enables biological experiments, such as near-simultaneous observation of two distinct brain layers, and clearer projection images inside complex, multi-cellular samples through blur removal, as only structures of interest are imaged. At the same time, props retains the advantages of conventional projection imaging, such as a traditional top-down view in OPM and LLSM, owing to the optical implementation of the shear warp transform.

The main technical innovation lies in leveraging the rolling shutter mode of scientific CMOS cameras for projection imaging. The rolling shutter mode has been used previously in light-sheet microscopy to improve optical sectioning, such as in digitally scanned light-sheet microscopy (DSLM)^11^, and to improve the trade-off between axial resolution and field of view in axially swept light-sheet microscopy (ASLM)^13^. In the context of confocal microscopy, rolling pixel readouts have been used as variable slit apertures^21–23^. In props, we instead use the rolling shutter to control the depth of a projection, which can exceed the depth of focus of LSFM and confocal microscopy by 1–2 orders of magnitude for wide shutter widths. In contrast, opening the rolling shutter in DSLM, ASLM, or line-scan confocal microscopes will not significantly increase the depth of focus of the overall imaging system. In the case of DSLM and ASLM, the depth of focus is governed by the light-sheet thickness and the detection optics and typically is on the order of a single micron.

Thus, it is important not to confuse our method with creating a confocal slice (see also Supplementary Fig. 9 for confocal imaging examples). Instead, props can be seen as an analogy to digitally rendering 3D data: the rolling shutter width decides “optically” how many and which planes should be included in a projection, similar to how graphics software allows a user to perform projections over a selected range of slices in a 3D data set. Props, however, provides such a projection view directly, i.e., it circumvents the 3D data acquisition and the post-processing step.

The upper limit of the projection depth is given by the light-sheet characteristics (i.e., its propagation length or confocal parameter, respectively), and as such, can be on the order of tens to hundreds of microns. The rolling shutter thereby also decides which signals to reject, leading to sharper images as highly scattering regions can be excluded from imaging and out-of-focus excitation is rejected. This considerably improves projection imaging inside complex, multi-cellular samples.

Selective projection imaging also enables opportunities for live cell super-resolution imaging, which typically faces the trade-off between spatial and temporal resolution. Props combined with structured illumination, as demonstrated here by using OPSIM, may allow the visualization of clathrin-mediated endocytosis or dynamics of the endoplasmic reticulum over selected layers within a cell. In contrast to rapid super-resolution imaging modalities such as TIRF- or GI-SIM, which are tied to the coverslip-cell interface, props may allow selective imaging of the peri-nuclear space or the dorsal membrane.

When props is combined with OPM, the axial position of the projection can be varied without mechanical motion of the sample or the objective, leading to rapid projection imaging of distinct sub-volumes. We have leveraged this to rapidly image two distinct layers in the zebrafish brain, which would not be possible at such rapid switching rates with conventional confocal or light-sheet microscopy due to the slower mechanical motion of a microscope stage or objective. We envision that this ability could be applied to superficial layers of the brain cortex in rodents or acute brain slices. While an ETL can also be used for refocusing, this will result in a slower settling time^24^ than with the galvo mirrors used here. Further, aberrations can be introduced^3^, and the integration of the ETL would require more changes to the microscope. Thus, our method represents an approach to implement such axial refocusing with higher speed and less complexity (only one galvo is added to the setup) while maintaining the microscope system’s native resolution.

In principle, it is possible to acquire even 3D stacks with props using narrow widths of the rolling shutter and applying small axial offsets sequentially (Supplementary Fig. 12 and Supplementary Movie 9). However, this trades off the ability of LSFM to acquire data in a parallelized manner. Acquiring multiple projections sequentially thus only makes sense if the total acquisition speed is faster than traditional 3D acquisition in LSFM.

The improved acquisition speed distinguishes props from real time de-skewing and rendering software that has been recently introduced for OPM^25^. Whereas computational rendering is limited by the speed a 3D stack can be streamed from the camera and stored in fast memory, props only need to acquire a single 2D image per time point. We explore these constraints on acquisition speed in Supplementary Note 4.

Besides speed, projection imaging also drastically reduces data size compared to 3D imaging, which can limit rapid, repeated, long time-lapse imaging of large samples in light-sheet microscopy. We imaged ventral furrow formation in a *Drosophila* embryo labeled with Gap43::mCherry over 100 timepoints in the projection mode (Supplementary Movie 10**)**, reducing the data size ~40-fold compared to conventional 3D stacking (Supplementary Note 4). As such, this allowed us to acquire numerous time-lapse series on different samples in projection mode to optimize experimental conditions before committing to a much more data-intense 3D acquisition run (Supplementary Fig. 13).

Overall, we believe these imaging capabilities will be beneficial for imaging experiments of rapid dynamics and multi-scale imaging approaches. As props can be implemented on existing camera-based microscopes such as light-sheet instruments without much modification, we anticipate that it will become a widespread feature of such instruments.

## Methods

The research within this work complies with all relevant ethical regulations as reviewed and approved by the University of Texas Southwestern Medical Center; the zebrafish work described in this manuscript has been approved and conducted under the oversight of the Institutional Animal Care and Use Committee (IACUC) at UT Southwestern under APN 2016-101805 to Gaudenz Danuser.

### Oblique plane microscopy

The images in the main manuscript were acquired with a recently published oblique plane microscope (OPM) with optical tiling capability^26^. The system was not physically changed compared to the published version, but the rolling shutter mode and advanced timing options were implemented in the control software. A description of the calibration of the shear galvo, an example of the drive signals, and an assessment of the linearity are discussed in Supplementary Note 5 and Supplementary Figs. 14–16. This calibration procedure was also applied to the other microscope used in this manuscript.

For the vasculature imaging in Fig. 1, a recently developed mesoscopic OPM system was used^14^.

### Lattice light sheet microscopy

We used a microscope system that can generate equivalents to dithered lattice light sheets through Field Synthesis^27^. In short, the system employs an NA 1.1 detection objective and an NA 0.7 illumination objective, the same lenses that have been used in the seminal LLSM publication^9^.

### Spinning disk confocal microscopy

A Nikon SoRa spinning disk microscope (CSU-W1 SoRa) was used, with a 40× NA1.3 oil objective (CFI Plan Fluor 40× Oil) for cellular imaging and a 40× NA1.15 water immersion objective (CFI Apo LWD Lambda S 40XC WI) for drosophila embryo imaging, respectively.

### Oblique planes structured illumination microscopy

We used our previously published Oblique plane structured illumination microscope (OPSIM) in a projection modality. To this end, a galvo unit was placed in front of the detection camera^16^. For the modification to props imaging, besides using a rolling shutter on the camera, offsets for the projection galvos were adjusted for each azimuthal orientation of the SIM pattern. These small offsets were needed to axially overlap each projection layer, which was verified on thin samples. We believe that the orientation-dependent offsets were necessary due to slight lateral shifts of the image rotator used in the OPSIM instrument. These shifts were not noticeable in the pro modality but became relevant when using props.

### *Drosophila* stock maintenance

*Drosophila melanogaster* stocks were kept in vials and bottles in 25 °C incubators. Both male and female flies were used in the study.

### Preparation of fly embryos for imaging actin and calcium sensor

*Drosophila* embryos expressing transgenes were generated using the Gal4/UAS system^28^. Transgenic fly lines that express Gal4 in the mesoderm (*Mef2-Gal4*) or carry *UAS-Actin5C-RFP* were acquired from the Bloomington *Drosophila* Stock Center. The *UAS-Actin5C-RFP* or *UAST-jGCaMP7s-CAAX* (generated in this study) transgenic flies were bred with the *Mef2-Gal4* flies. Embryos were collected by grape juice agar plates for 4–12 h at 25 °C, dechorionated in 50% bleach for 3 min, rinsed with water, and aligned onto a glass bottom MatTek dish. To avoid rolling and drifting of the embryo, a heptane glue (double-sided tape (3 M) dissolved in heptane) coated coverslip was placed on top of the embryo. Subsequently, embryos were covered with a thin layer of Halocarbon oil (Sigma) to keep them alive.

### Preparation of fly embryos for imaging ventral furrow formation

A 90-min collection of *Drosophila* embryos expressing *Sqh*>Gap43::mCherry (gift from A. Martin) and SqhFRB-GFP; +; gap43mCherry/TM3 were aged for 90–120 min at 25 °C. Embryos were dechorionated, oriented, and mounted ventral side down on a glass bottom MatTek dish (P35G-1.5-14 C, Mattek).

### Mounting the zebrafish embryo

To image the zebrafish vasculature with OPM or mesoOPM, the zebrafish were embedded in 0.5–1% low melting agarose on a glass bottom MatTek dish. Before the agarose was gelled, we added a coverslip on top of the liquid agarose, and this helped to push the zebrafish close to the bottom of the MatTek dish to ensure the zebrafish embryo was within the working distance of the objective.

To image the brain activity of zebrafish with an OPM, the zebrafish not only needs to be close to the bottom of the MatTek dish but also needs to be flipped to make sure the brain is facing down. To do so, with the mounting protocol described previously, we also quickly flip the MatTek dish before the agarose is gelled. This is simply because the zebrafish usually keep their heads up in a natural condition, while flipping the MatTek dish, they tend to orientate themselves so now their heads are close to the glass of the MatTek dish. Therefore, when we image through the bottom of the MatTek dish with OPM or other inverted microscopes, their brains face down.

### Fly genetics and molecular biology

UAST-jGCaMP7s-CAAX plasmid was generated through Gibson cloning. jGCaMP7s (Addgene plasmid # 104463^29^;) and CAAX nucleotide sequences were PCR-amplified from pGP-CMV-GCaMP6s-CAAX (Addgene plasmid # 52228^30^;), using the primer pairs as follows:

jGCaMP7s_FW: aagagaactctgaatagggaattgggaattcatgggttctcatcatcatcatc jGCaMP7s_RV: catcttttcttttgctcctgctcccttcgctgtcatcatttgtacaaac CAAX_FW: gtttgtacaaatgatgacagcgaagggagcaggagcaaaagaaaagatg CAAX_RV: agtaaggttccttcacaaagatcctctagattacataattacacactttgtctttg

The resulting PCR amplicons were then cloned into the *Drosophila* transformation vector pUASTattB^31^ between EcoRI and XbaI restriction sites using Gibson Assembly. Subsequently, a transgenic fly strain carrying UAST-jGCaMP7s-CAAX at the attP2 site was generated by ΦC31 integrase-mediated germline transformation (Rainbow Transgenics).

The fly line carrying GFP-tagged myosin and membrane-Cherry was previously described^32^. In brief, the gap43-Cherry construct on the third chromosome^33^ was combined with a non-muscle myosin regulatory light chain tagged with FRB-GFP at the endogenous locus.

### Zebrafish husbandry

Zebrafish work described in this manuscript has been approved and conducted under the oversight of the Institutional Animal Care and Use Committee (IACUC) at UT Southwestern under APN 2016-101805 to Gaudenz Danuser.

Zebrafish (*Danio rerio*) adults, embryos, and larvae were kept at 28.5 °C and were handled according to established protocols^34^. To visualize zebrafish vasculature, we imaged zebrafish expressing the vascular marker *Tg(kdrl:Hsa.HRAS-mCherry)*^35^ or Tg(*kdrl:EGFP*)^36^ in a Casper background^37^. To study neuronal firing in zebrafish larval brains, we imaged zebrafish expressing the calcium marker Tg(*elavl3:soma-GCaMP7f*)^20^. To induce neuronal activity, we added 800 μM 4-aminopyridine (4-AP) (Sigma A78403) to the egg water during imaging as previously described^38^. All zebrafish experiments were performed at the embryonic and larval stages, and therefore the sex of the organism was not yet determined.

### Statistics and reproducibility

Given a lack of expectations for effect size before the experiments were performed, no a priori power analyses were conducted to determine adequate *n*-values. For each experiment, we therefore, only made sure to perform at least *n* = 3 repeats to ensure technical replicability. Randomization and blinding were not applicable in this study and were not performed.

### Reporting summary

Further information on research design is available in the Nature Portfolio Reporting Summary linked to this article.

### Supplementary information

Supplementary Information

Reporting Summary

Description of Additional Supplementary Files

Peer Review File

Supplementary Movie 1

Supplementary Movie 2

Supplementary Movie 3

Supplementary Movie 4

Supplementary Movie 5

Supplementary Movie 6

Supplementary Movie 7

Supplementary Movie 8

Supplementary Movie 9

Supplementary Movie 10

### Source data

Source Data file

## Data Availability

The image and movie data generated in this study have been deposited in a public Zenodo repository: https://zenodo.org/records/10182659 Source data are provided with this paper.

## Code availability

The Matlab and Python codes used for data analysis are available in a public GitHub repository. The LabView-based control software used within this manuscript to control the OPM and LLSM systems is freely available for nonprofit institutions through a Material Transfer Agreement with UT Southwestern Medical Center.
