# Supplementary material for: Projective light-sheet microscopy with flexible parameter selection
Source: Nat Commun. 2024 Mar 29;15:2755. doi: 10.1038/s41467-024-46693-y (PMC10980818; doi:10.1038/s41467-024-46693-y)
Supplement: Supplementary file 1 — Supplementary Information [file 41467_2024_46693_MOESM1_ESM.pdf]

Supplementary Information

Projective light-sheet microscopy with flexible parameter selection

Authors

Bingying Chen[†1], Bo-Jui Chang[†1], Stephan Daetwyler[1], Felix Zhou[1], Shiv Sharma[2], Donghoon M. Lee[2], Amruta Nayak[3], Jungsik Noh[1], Konstantin Dubrovinski[4,5], Elizabeth H. Chen[2,5,6], Michael Glotzer[3], Reto Fiolka[1,5]

Affiliation

[1]Lyda Hill Department of Bioinformatics, University of Texas Southwestern Medical Center, Dallas, TX, USA.

[2]Department of Molecular Biology, University of Texas Southwestern Medical Center, Dallas, TX, USA.

[3]Department of Molecular Genetics and Cell Biology, University of Chicago, Chicago, IL, USA

[4]Department of Biophysics, University of Texas Southwestern Medical Center, Dallas, TX, USA.

[5]Department of Cell Biology, University of Texas Southwestern Medical Center, Dallas, TX, USA.

[6]Hamon Center for Regenerative Science and Medicine, University of Texas Southwestern Medical Center, Dallas, TX, USA

Correspondence

Correspondence to Reto Fiolka: reto.fiolka@utsouthwestern.edu

[†]These authors have contributed equally to the manuscript

Supplementary Figures

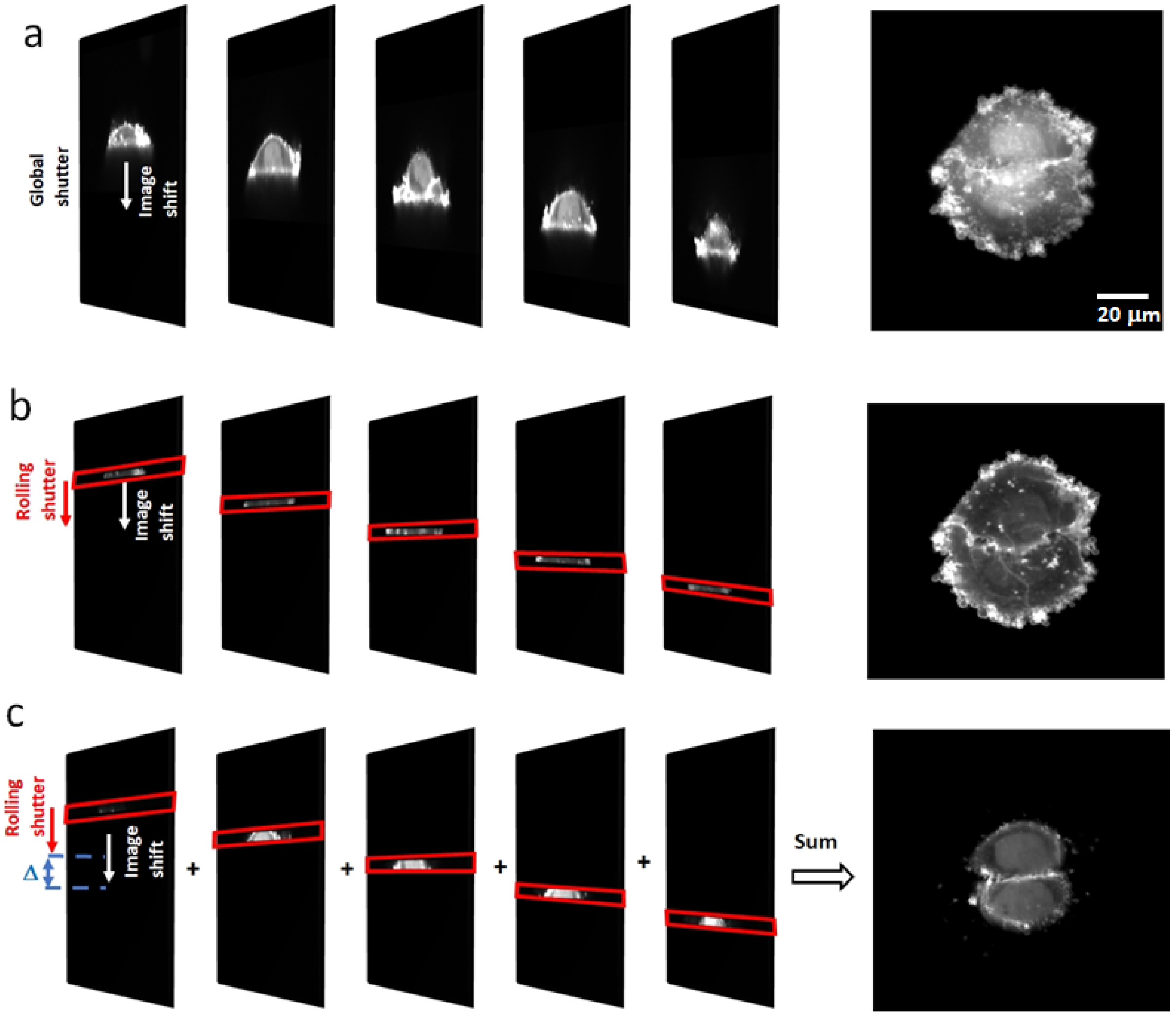

Supplementary Figure 1. Numerical simulation of different projection modalities on a 3D data stack. a Numerical simulation of a projection using global shutter by summing the planes of a conventional 3D stack (a shift to each image frame was added numerically). The resulting projection is shown on the right. **b** Simulation of props. Outside of the red box, all values are set to zero in the stack. The red box moves with the numerical image shift. The resulting projection is shown on the right. **c** Simulation of an axially shifted projection. The red box is shifted upwards in the image frame. The resulting projection is shown on the right. The raw data for this simulation was from a MV3 cell labeled with AKT-PH-GFP acquired with a Field Synthesis light-sheet microscope as a conventional z-stack.

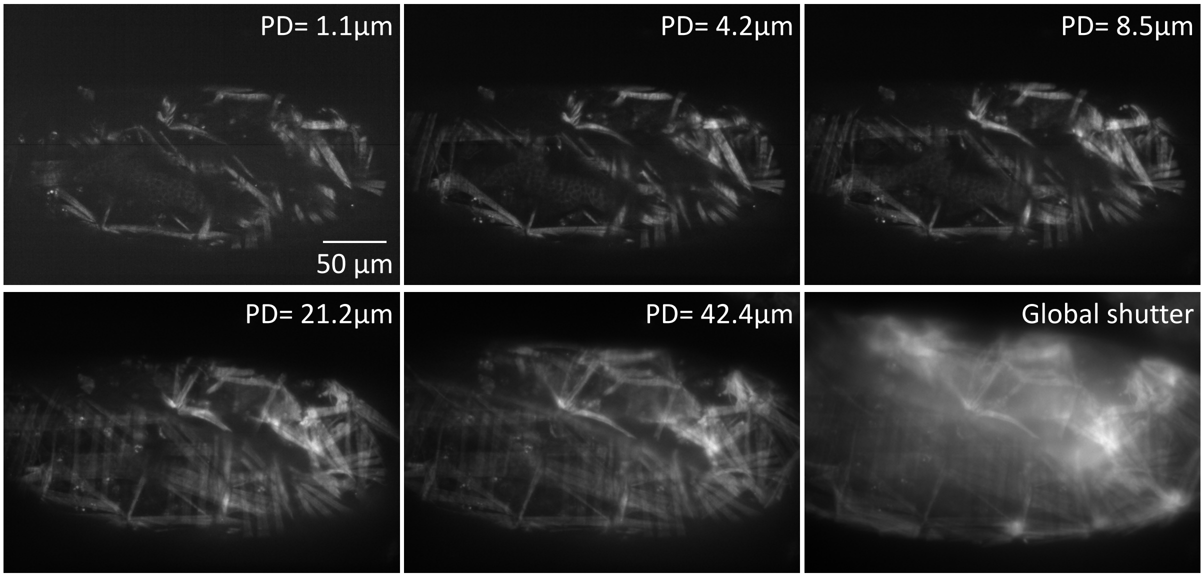

Supplementary Figure 2. Variation of the projection depth. Drosophila embryo, labeled with actin5C-RFP, as imaged with different rolling shutter width (resulting projection depth, PD, indicated in microns). Bottom right image shows a projection using global shutter.

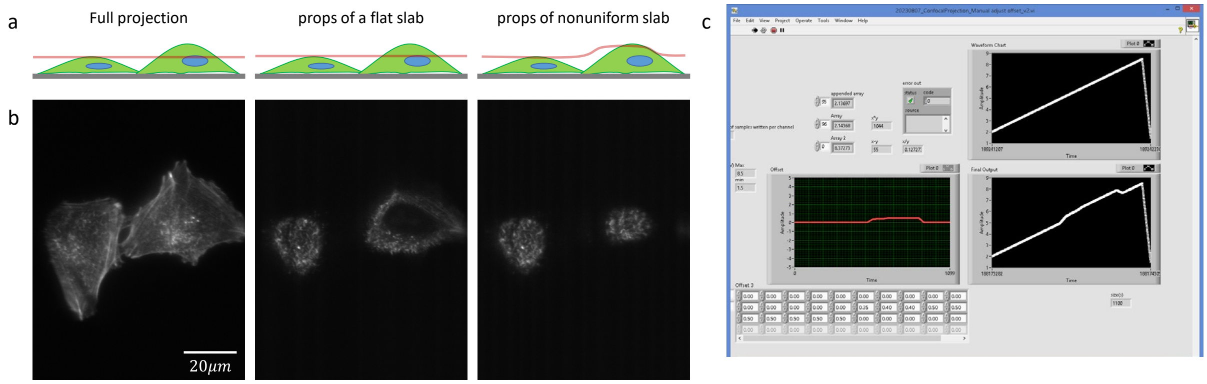

Supplementary Figure 3. Projection imaging of a nonuniform sub-volume with props. **a** Conceptual sketch of how props can selectively image the top of both cells located at different heights above the coverslip. **b** Projection image of two cells imaged with full projection, a flat slab, and a nonuniform slab. **c** Screenshot of the control software. The red curve is similar to the red line in a, which fits the height of the two cells. The white curve on the lower right is the final output signal to the scanning- and shear-galvos.

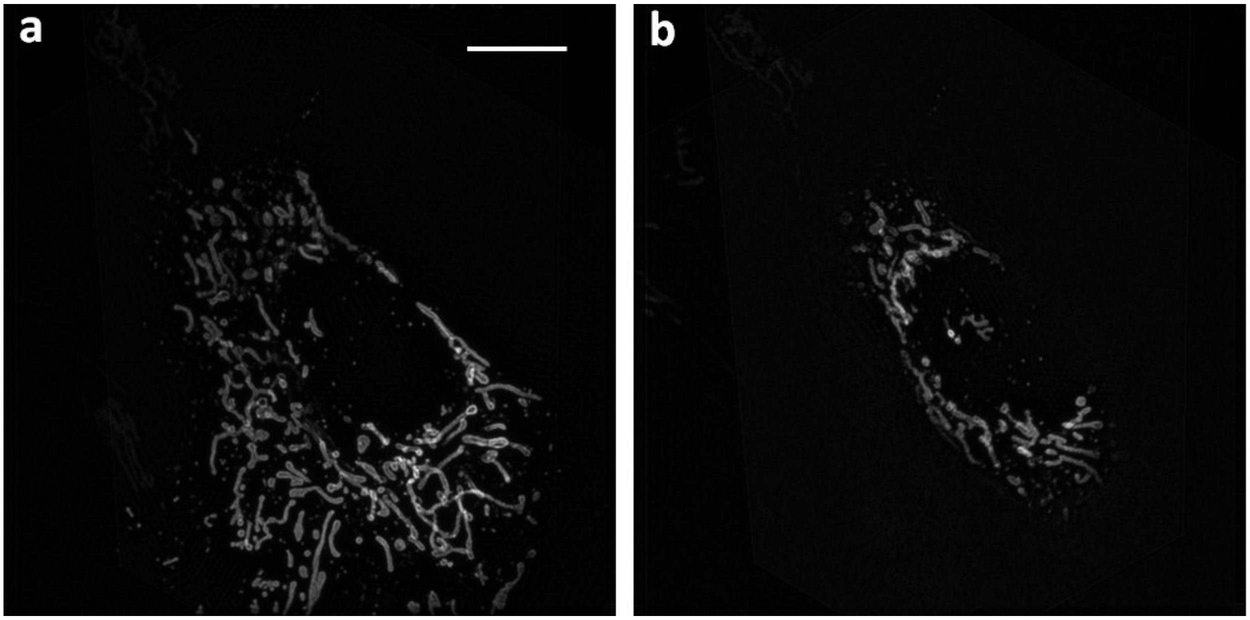

Supplementary Figure 4. Projection imaging in structured illumination microscopy. a, b U2OS cells, labeled with OMP-GFP, as imaged with props using oblique plane structured illumination microscopy. The separation between the two planes (**a, b**) is 4 microns, and the thickness of each projection is 2.5 microns. Scale Bar: 10 microns.

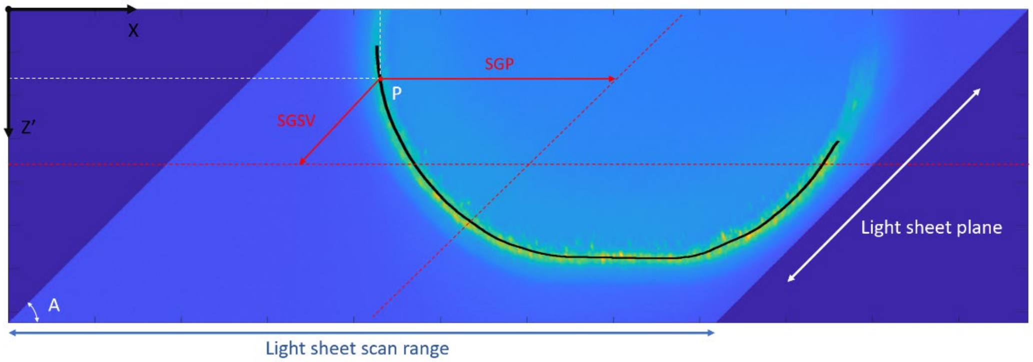

Supplementary Figure 5. Definition of a surface scanning curve. A side view (x-z') of a 3D stack containing a Drosophila embryo is shown. The OPM scans a light-sheet along the x-direction, which samples a parallelogram shaped volume (light blue). The cells at the surface of the embryo are shaded in light blue to yellow. A black line depicts the target curve with which the light-sheet and the shear galvo will be scanned such that the content along that curve is mapped onto the rolling shutter. SGP: Scanning Galvo Position; this signal controls the position of the light-sheet in the sample plane. SGSV: Shearing Galvo Shift Value: this controls where the image from the light-sheet is positioned on the camera detector. Light sheet scan range: 245 microns.

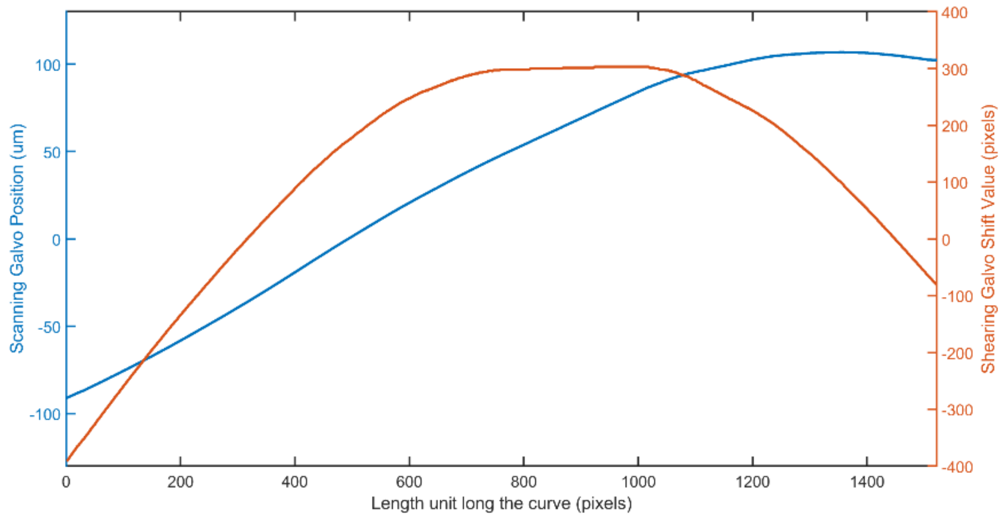

Supplementary Figure 6. Example curve in the side view. Values for Scanning Galvo Position and Shearing Galvo Shift Value along the curve shown in **Supplementary Figure 5**. Source data are provided as a Source Data file.

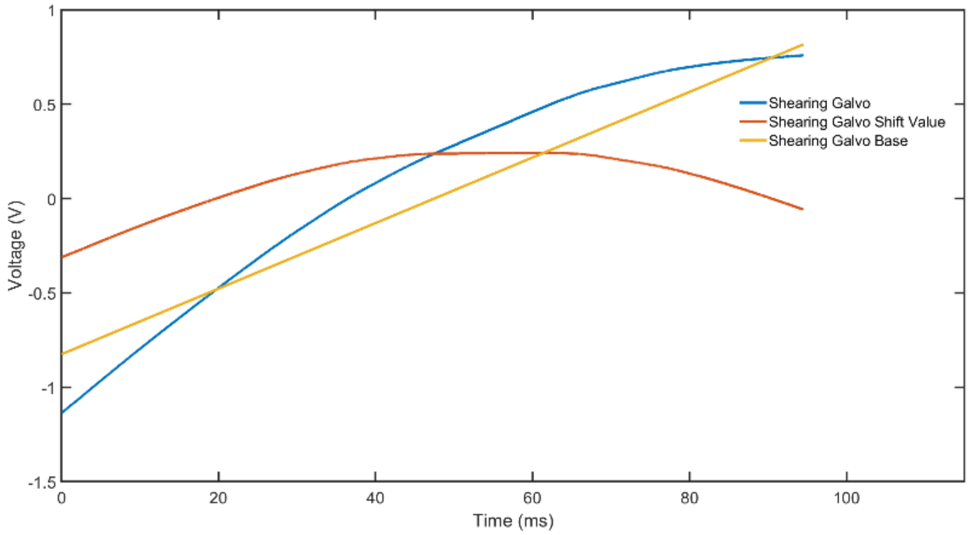

Supplementary Figure 7. Example control voltage of the shearing galvo. Voltage signals for the Shearing galvo (blue), which is the sum of Shear Galvo Base and Shearing Galvo Shift Value. Source data are provided as a Source Data file.

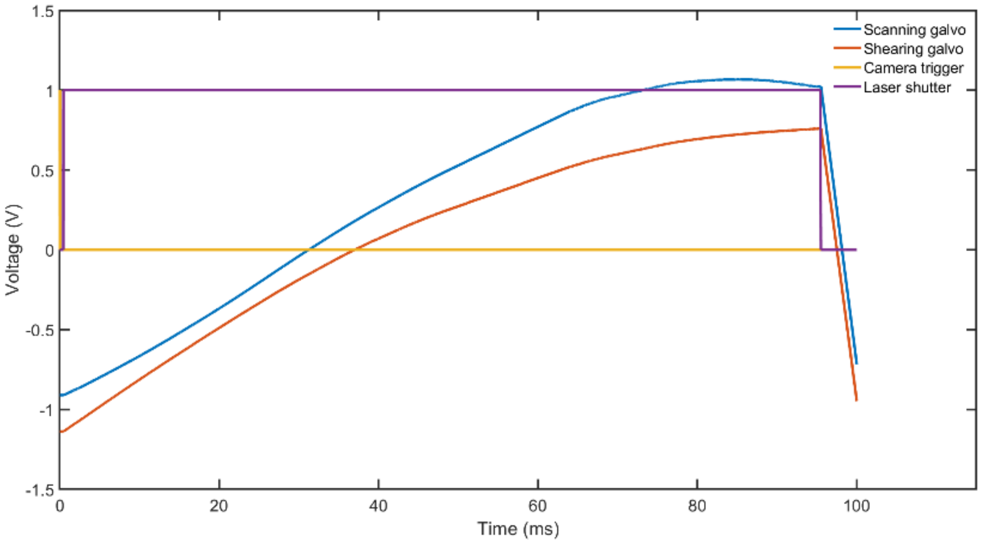

Supplementary Figure 8. Control Waveforms for a curved projection. Drive signals for the shear galvo, the OPM scanning galvo, the camera trigger, and the laser shutter, which were used to acquire the curved projection shown in **Figure 2i**. Source data are provided as a Source Data file.

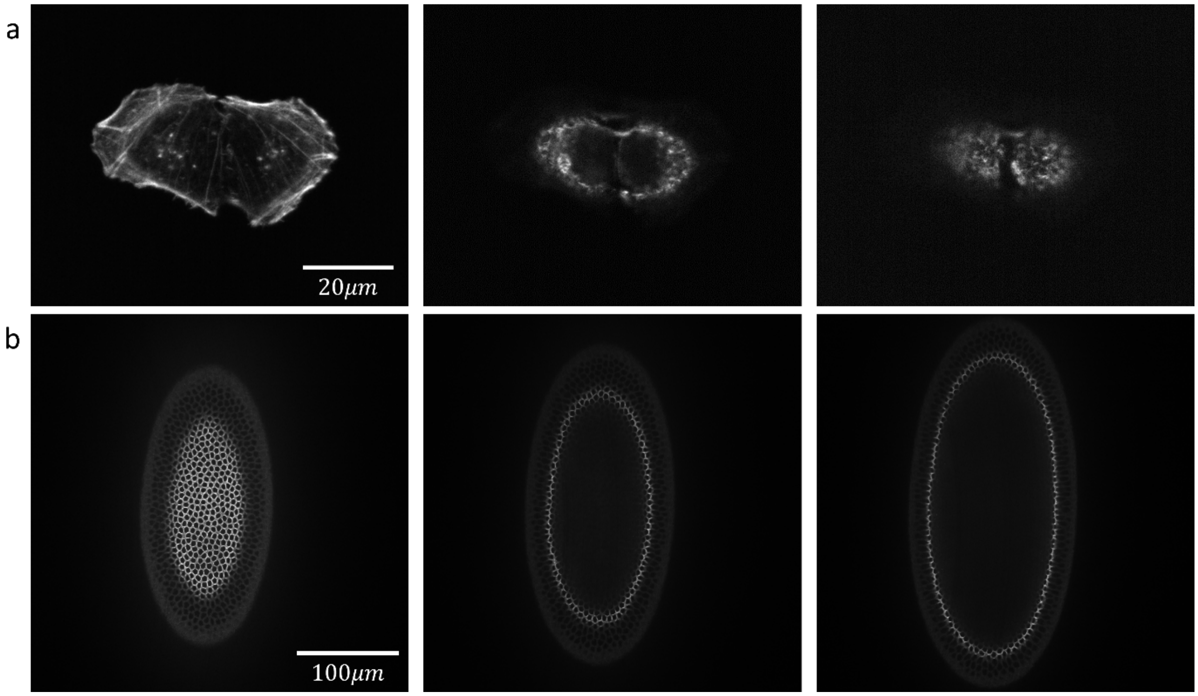

Supplementary Figure 9. Representative spinning disk confocal images of a single cell and a Drosophila embryo. a Three confocal slice through a A375 cells labeled with F-tractin-EGFP, acquired with a commercial spinning disk confocal microscope (CSU-W1 SoRa, Nikon) with a 40x NA1.3 oil objective (CFI Plan Fluor 40X Oil). **b** Three confocal slices through a Drosphila embryo labeled with myosin-GFP using the same spinning disk microscope with a 40X NA1.15 water immersion objective (CFI Apo LWD Lambda S 40XC WI).

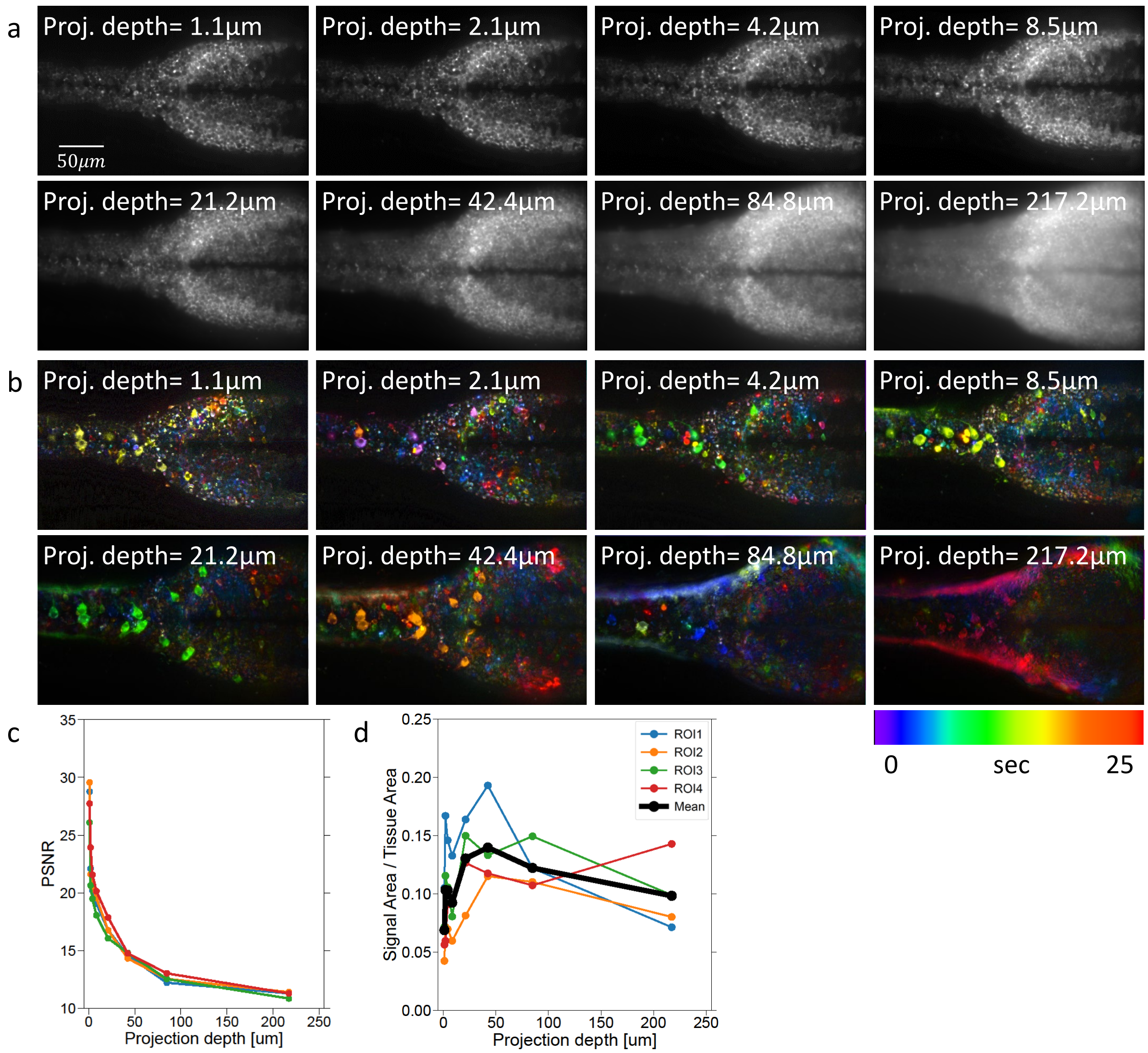

Supplementary Figure 10. Effect of projection depth in calcium imaging. a The average temporal projection of the timelapse at different projection depths. **b** The temporal color-coded maximum intensity projection of the timelapse after the average of the entire time series has been subtracted. **c** Quantification of the image contrast using PSNR. **d** Quantification of the number of pixel firing events at different projection depths. Please note that 4 datasets acquired from the same fish are plotted in c and d, whereas in a and b only one dataset (ROI1) is presented as an example. Source data are provided as a Source Data file.

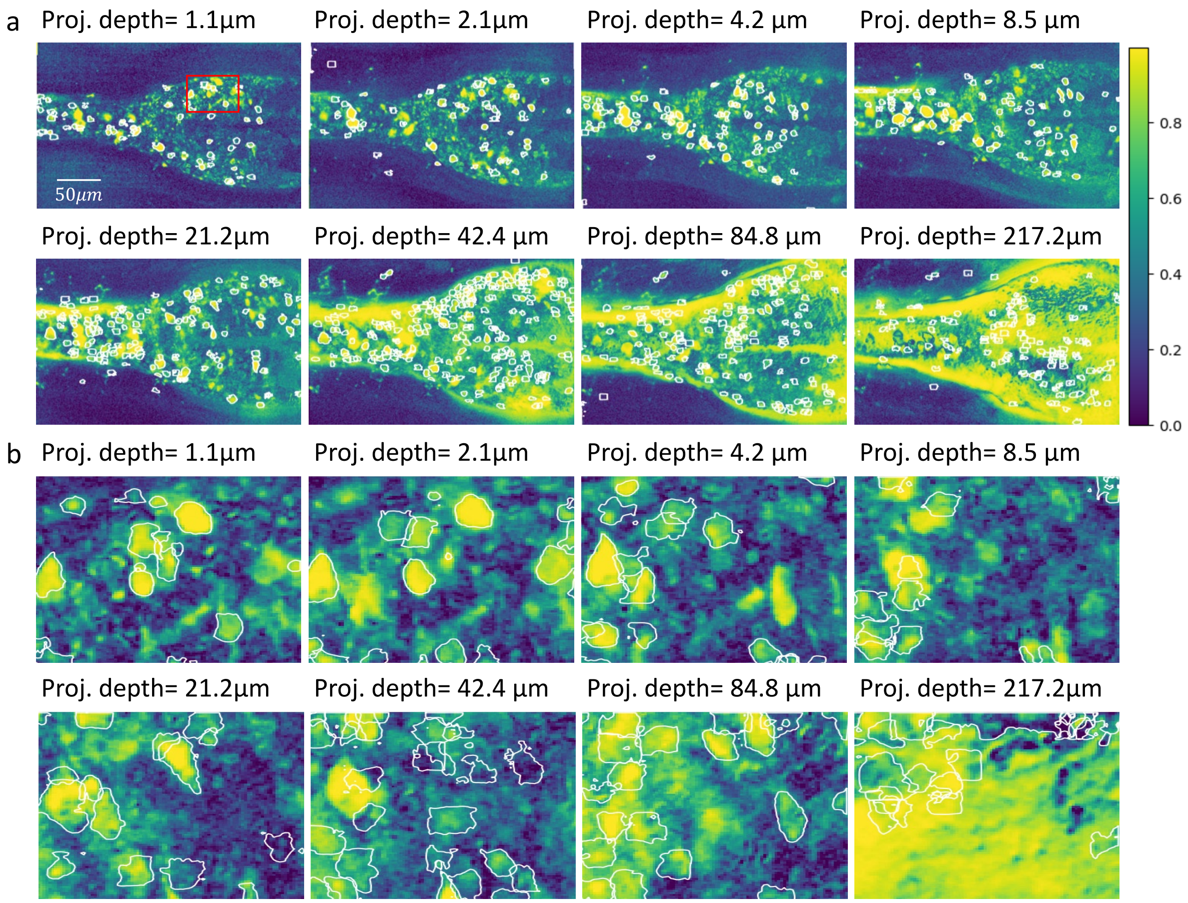

Supplementary Figure 11. Quantification of firing neurons with CaImAn. **a** The correlation image with the annotated ROIs at different projection depths. **b** The zoom-in of the red box in **a**.

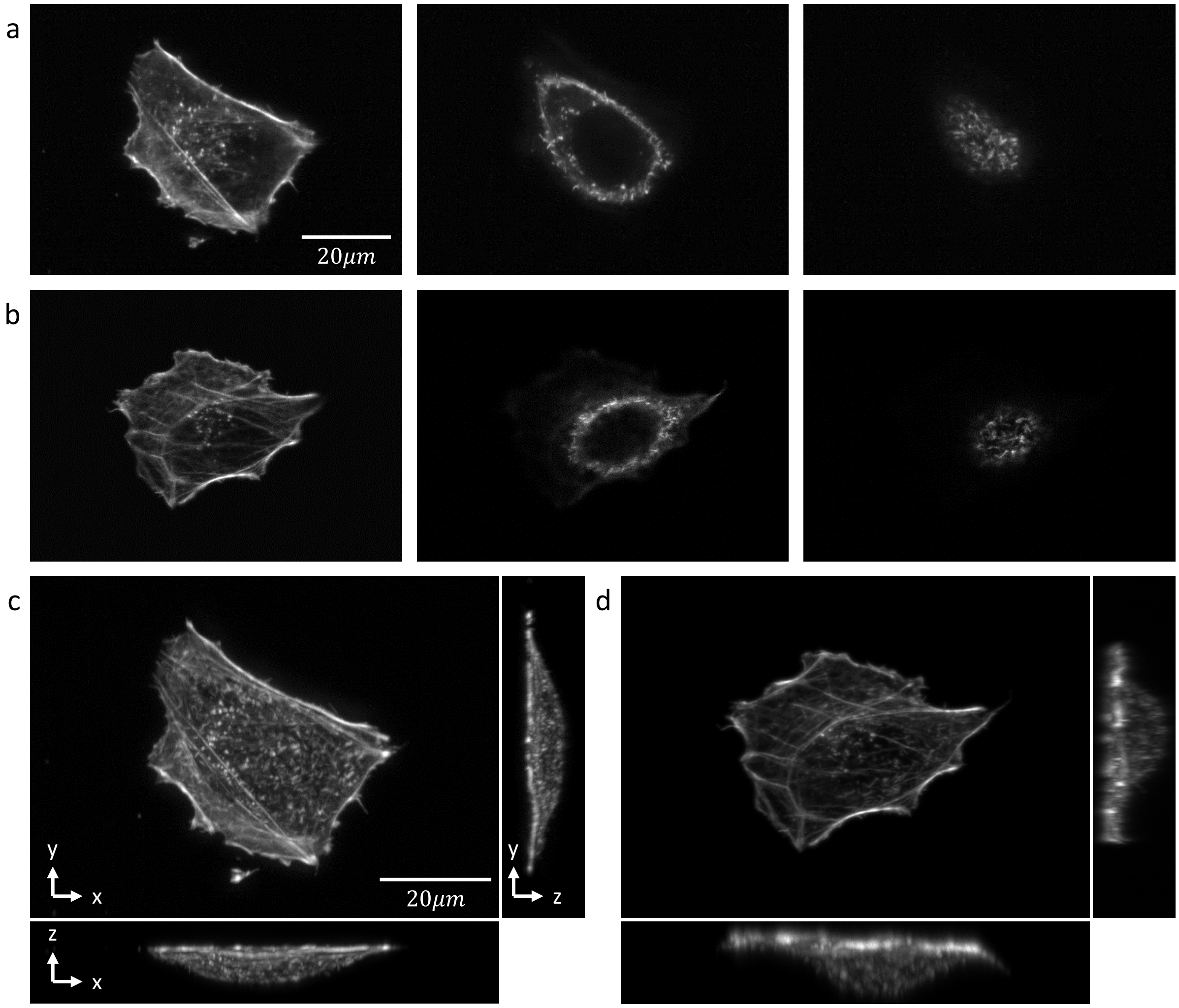

Supplementary Figure 12. Comparison of a 3D stack assembled from a series of projections, and one acquired with a spinning disk confocal microscope. Fixed A375 cells labeled with F-tractin-EGFP were used for the imaging. **a** A series of projections acquired with a Field Synthesis light-sheet microscope, where the axial position of each projection was stepped through the sample. **b** A series of confocal slices acquired with a commercial spinning disk confocal microscope (CSU-W1 SoRa, Nikon) using a 40x NA1.3 oil objective (CFI Plan Fluor 40X Oil). **c** and **d** The maximum intensity projections in XY, XZ, and ZY of **a** and **b**, respectively.

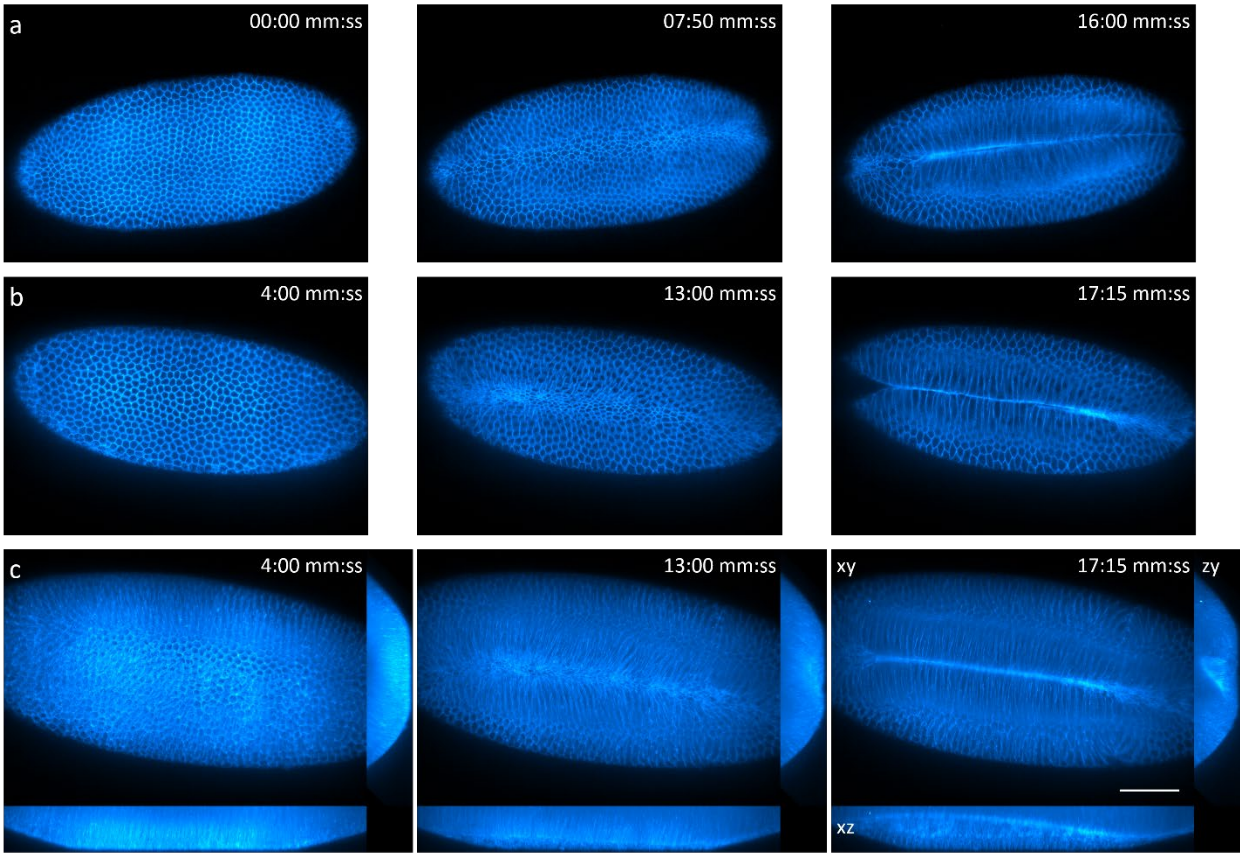

Supplementary Figure 13. Comparison of projections to numerical projections obtained from a 3D stack. a Three time points from a time series of projections from a Drosophila embryo labeled with Gap::mCherry using OPMprops. **b** Three time points from another embryo at similar stages of development, but using volumetric imaging (i.e. a 3D Stack per timepoint was acquired with OPM) and numerically projecting the data over a similar depth as in **a**. **c** Maximum intensity projections from the same 3D stacks as in **b**, but projected over the whole depth of the stack. Orthogonal views are shown below and on the side. Scale bar: 50µm.

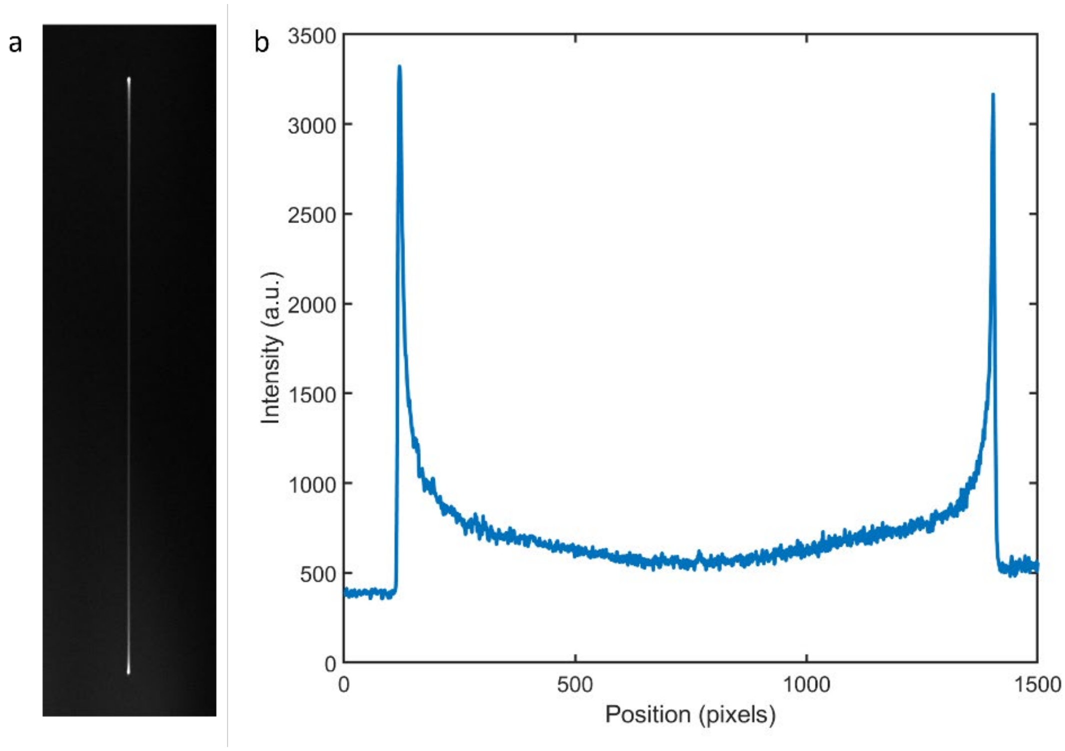

Supplementary Figure 14 Shear galvo calibration. a the image of a scanning laser spot on the camera, which is dynamically scanned with the shear galvo. **b** the profile along the line in a. Source data are provided as a Source Data file.

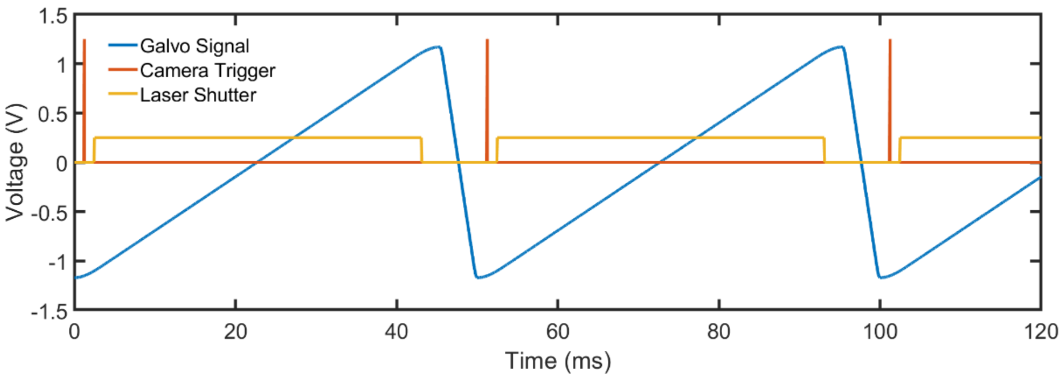

Supplementary Figure 15. Example control signals for props. The blue galvo signal drives the OPM scanning galvo, and a scaled version of the signal is applied to the shearing galvo. Source data are provided as a Source Data file.

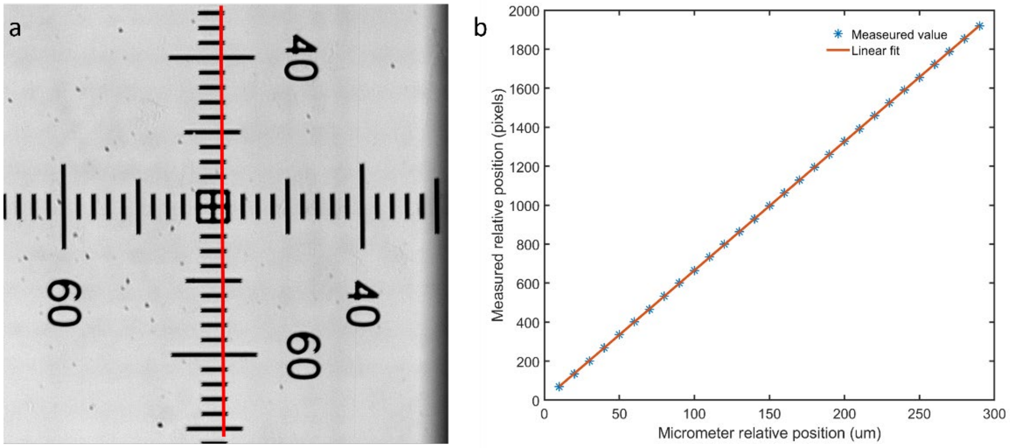

Supplementary Figure 16. Assessing the linearity of a projection image. a Microruler as imaged with OPMprops**.** The shear direction runs vertically. **b** Comparison of micrometer increments to projection pixel distances along the red curve in **a**. Source data are provided as a Source Data file.

Supplementary Notes

Supplementary Note 1: Props can provide a projection image of a nonuniform sub-volume.

Props can vary the axial position of where the projection image is formed. Moreover, the axial position can be spatially varied across the same field of view (FOV) in one camera exposure. **Supplementary Figure 3** shows two adjacent cells, each of different heights. With props, we can image a thin slab of the cell. If we adjust the axial position of the slab to the top of the short cell uniformly over the FOV, the projection image of the tall cell will appear hollow in the middle. However, by varying the axial position of the slab nonuniformly we can now image the top of both cells in the same FOV. This is a unique feature of props. To our knowledge, no other available common microscopy techniques such as confocal or spinning disk confocal allow for nonuniform variation of imaging plane in the same FOV.

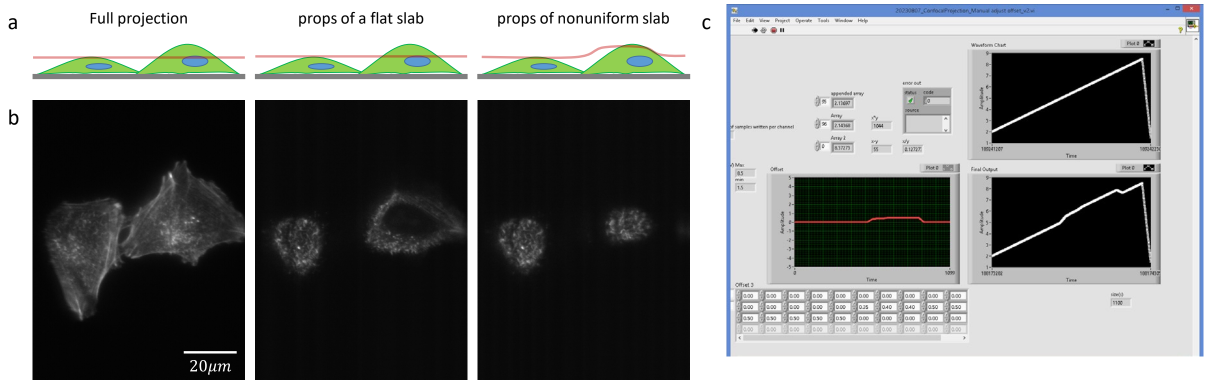

Supplementary Figure 3. Projection imaging of a nonuniform sub-volume with props. **a** Conceptual sketch of how props can selectively image the top of both cells located at different heights above the coverslip. **b** Projection image of two cells imaged with full projection, a flat slab, and a nonuniform slab. **c** Screenshot of the control software. The red curve is similar to the red line in a, which fits the height of the two cells. The white curve on the lower right is the final output signal to the scanning- and shear-galvos.

The projection image of a nonuniform slab can be easily done as follows. To acquire a projection image, a ramp signal (shown on the upper-right in Supplementary Figure 3c) is applied to both the scanning galvo and shear galvo. To vary the axial position of the projection image, an offset voltage just needs to be added to the ramp signal. Therefore, to adjust the axial position nonuniformly over the FOV, we just need to add a nonuniformly offset voltage, which corresponds to the desired axial position, e.g. the height of two cells here. As shown on the lower right in Supplementary Figure 3c, the final output signal is simply the sum of the red curve and the normal ramp signal (white curve on the upper right in Supplementary Figure 3c.

Supplementary Note 2: Procedure to calibrate waveforms for curved projection imaging.

In this note we explain how we form projections along tilted and curved surfaces. In the projection imaging mode, when the rolling shutter is set to a narrow width, a thin projection along the lateral plane will be acquired. By changing the offset of the control signal, we can shift the imaging plane in Z dimension. This can happen in discrete steps for a few scans, resulting in image planes at different z-planes. Or if the offset changes continuously during a scan, a tilted or curved surface can be acquired. Therefore, we can design an offset function to acquire a curved plane in a volume.

As a practical example, we show the steps to unwrap the surface of a Drosophila embryo. **Supplementary Figure 5** shows a cross-section through a volumetric acquisition of an embryo, which allows us to compute a waveform that will result in a curved projection of its surface. To this end, we let the scanning galvo and shear galvo scan along the curve (the black line in **Supplementary Figure 5**) and map the content along it onto the linearly moving rolling shutter.

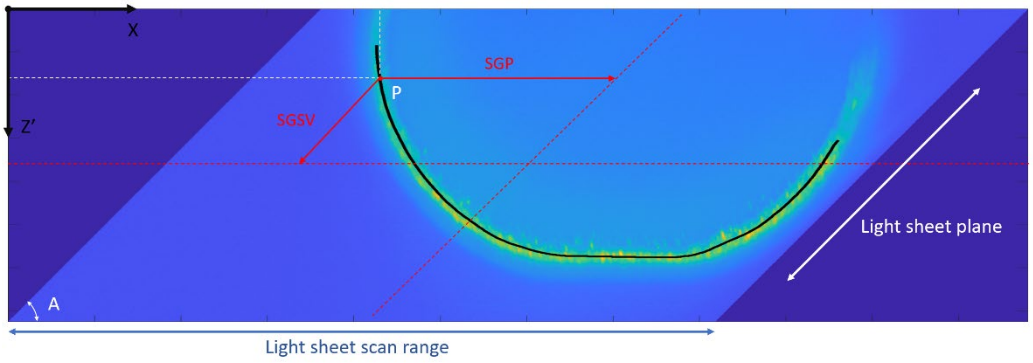

Supplementary Figure 5 Definition of a surface scanning curve. A side view (x-z') of a 3D stack containing a Drosophila embryo is shown. The OPM scans a light-sheet along the x-direction, which samples a parallelogram shaped volume (light blue). The cells at the surface of the embryo are shaded in light blue to yellow. A black line depicts the target curve with which the light-sheet and the shear galvo will be scanned such that the content along that curve is mapped onto the rolling shutter. SGP: Scanning Galvo Position; this signal controls the position of the light-sheet in the sample plane. SGSV: Shearing Galvo Shift Value: this controls where the image from the light-sheet is positioned on the camera detector.

To determine the desired scanning curve from the data, we first acquired a stack of the imaging volume to cover the region of interest. This stack was subsequently sheared and resampled to form a side view with isotropic pixel size. The side view was maximum intensity projected into 2D and one example is shown in **Supplementary Figure 5**. To match with the image coordinate system used in MATLAB, we adopted the coordinate convention (X, Z') as shown to represent the points on the curve. Z' equals to -Z in Figure 1. The tilted and horizontal red dash lines are the light sheet plane/Image plane with Scanning Galvo at 0 and the middle plane of the image volume.

We segmented the side-view image with the intensity threshold automatically determined by a multilevel Otsu method [1]. A four-level binary segmentation was performed, and we kept the highest intensity segmented region. This initial binary segmentation was then morphologically processed, keeping only the largest connected component. Since in this example, the intensity of the curved surface was distinctly

brighter than the rest of the embryo such that it corresponded to the highest intensity Otsu segmentation region, we could directly extract the scanning curve as the skeleton of the segmentation region. To extract a smooth skeleton with no side branches, we performed binary closing with disk structural element of radius 10 pixels, Gaussian filtered with sigma=35 pixels and rebinarized at a threshold of 0.1. The skeleton image corresponding to the final scanning curve was then computed using the bwskel MATLAB function, and bwboundaries MATLAB function was used to determine the coordinates.

We treated each point on the curve as a time point when scanning the curve. To traverse the curve at even step size, we resampled the curve according to the curve length and obtained the coordinates of each point to sample on the curve as (x, z'). According to the geometry before and after shearing, the Shearing Galvo Shift Value (SGSV) and SGP (SGP) can be determined as

$$\mathrm{SGSV} = (z' - \mathrm{mImage}/2)\ /\sin\,(A)$$

$$\mathrm{SGP} = [x - (\mathrm{mImage} - z')/\tan\,(A)] \cdot \mathrm{pixelsize} + \mathrm{SGP}_0$$

where A is the angle between the light sheet plane and scanning direction, mImage is the pixel number in Z', SGP_0 is the starting position of the scan range. SGSV and SGP's values along the curve are shown in **Supplementary Figure 6**. We set the unit as µm for SGP, while keeping the unit for SGSV as pixel for convenience in the next step.

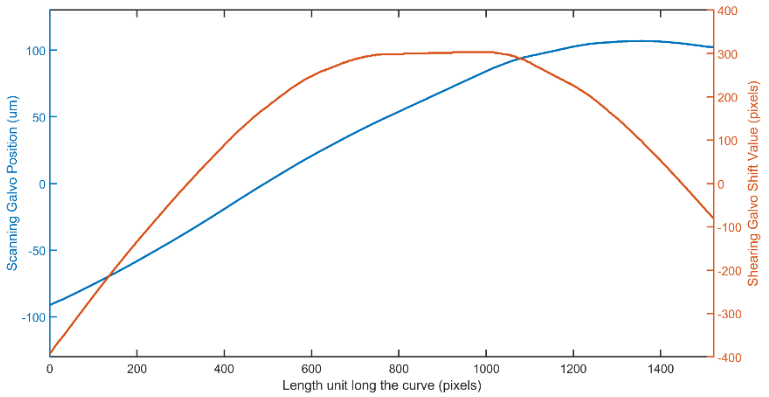

Supplementary Figure 6 Example curve in the side view. Values for Scanning Galvo Position and Shearing Galvo Shift Value along the curve shown in **Supplementary Figure 5**. Source data are provided as a Source Data file.

With the curve length in pixel unit and the step size as the pixel size, we could get the corresponding length of the curve in µm by multiplying curve length and pixel size. Also because of that, we set the camera's vertical lines as the rounded curve length in pixel unit (H). With H and exposure time (t_{expo}), the Shearing Galvo Base are formulized as

$$\text{Shearing Galvo Base} = H/t_{expo} \cdot t - H/2.$$

After measuring shear galvo voltage per pixel shift on the camera, we can have the Shearing Galvo control signal as the sum of Shearing Galvo Base and the Shearing Galvo Shift Value, shown in **Supplementary Figure 7**

$$\text{Shearing Galvo} = \text{Shearing Galvo Shift Value} + \text{Shearing Galvo Base}.$$

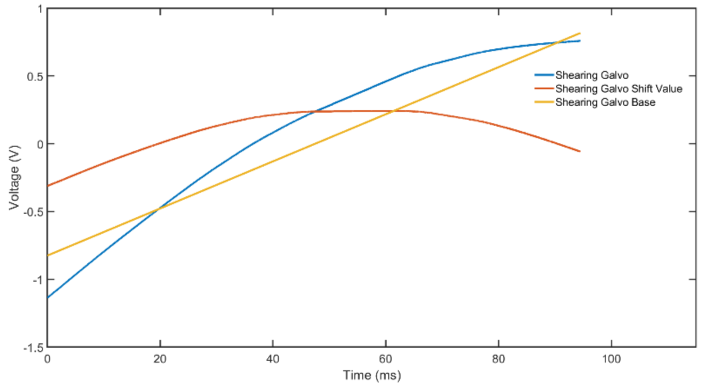

Supplementary Figure 7 Example control voltage of the shear galvo. Voltage signals for the Shearing galvo (blue), which is the sum of Shear Galvo Base and Shearing Galvo Shift Value. Source data are provided as a Source Data file.

Because the rolling shutter moves along the camera and the light sheet scans the curved plane at a constant speed and within one exposure, control signals as shown in **Supplementary Figure 8** result for the curved projection. The camera runs on External trigger mode (Edge trigger) with Camera trigger in the figure as input. These are the waveforms that were used to acquire the curved plane shown in **Figure 2i** within a frame.

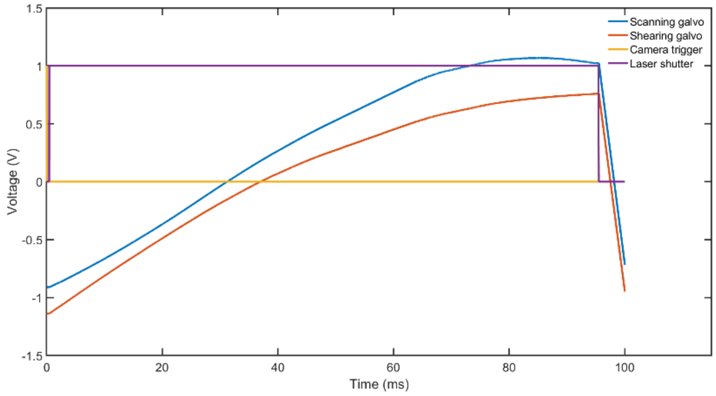

Supplementary Figure 8 Control Waveforms for a curved projection. Drive signals for the shear galvo, the OPM scanning galvo, the camera trigger, and the laser shutter, which were used to acquire the curved projection shown in **Figure 2i**. Source data are provided as a Source Data file.

Supplementary Note 3: Optimization of the projection depth for calcium imaging

In **Figure 3i-k** we show the calcium imaging in zebrafish brain from two distant sub-volumes ("slabs"). Due to the dense labeling, we have further optimized the projection depth to balance the amount of information in each projection and the signal to background ratio. This optimization is explored in more detail in this note.

Neurons vary heterogeneously in size and shape across the brain. Imaging with a confocal slice using a spinning disk or a thin projection with props may not capture the firing of the whole neuron. Meanwhile, a full 3D stack acquisition might be too slow to capture fast-firing dynamics. Since the projection depth can be readily changed in props, we investigated how projection depth affects the characteristics of the imaged calcium dynamics. We imaged calcium firings from the same region of the zebrafish brain with different projection depths. As shown in **Supplementary Figure 10a**, a thinner projection depth reduces blurring and leads to enhanced contrast. We quantified this observation by computing the peak signal-to-noise ratio (PSNR) with respect to a reference image defined by the maximum temporal projection of the timelapse taken at the thinnest projection depth (1.1µm) (**Supplementary Figure 10c**). However, with a thin projection depth, the number of firing events that are captured is reduced. We hypothesized because the size of the cell body of the average zebrafish neuron on the order of 10 µm, a thin slice may not even capture a single neuron layer. By increasing the projection depth, we may encompass entire neurons, which would increase detection of firing events. With even larger projection depths, multiple layers of neurons and their associated calcium signaling might be captured, at the expense of reduced signal to background ratio and more image blurring. Therefore, there we assume that there is an optimal projection depth range, which balances the number of firing events and imaging contrast. In **Supplementary Figure 10b**, we subtracted the average of the entire time series from each time frame and generated a temporal color-coded maximum intensity projection over time. As shown, although a thin projection depth (~1.1-4.2µm) provides sharper images, dotted structures are visible, which might be due to noise or capturing a portion of actual neurons. With a thicker projection depth (> 80µm), the periphery of the brainstem starts to appear. While multiple neurons are detected within this region, they are almost impossible to be individually segmented. It also becomes more challenging to segment single neurons in the center because of the high cell density.

Therefore, to quantify the number of firing events with respect to projection depth in an unbiased manner without segmenting individual neurons, we measured the total number of firing image pixels over the total number of image pixels corresponding to each projection depth (**Supplementary Figure 10d**). For each timelapse, after preprocessing (described below), individual frames were contrast stretched by clipping intensities to between 2nd and 99.8^{th} percentile. Intensities were then normalized by subtracting a background intensity, which was estimated by downsampling the frame by a factor of 16 and gaussian smoothing with σ=5 pixels. After renormalizing the intensity values to 0-1 using contrast stretching, the timeseries of individual pixels were smoothed using a moving median with time window of 11 frames. The autocorrelations of individual pixel timeseries were then computed. A periodic firing pattern induces oscillation in the autocorrelation. For each pixel timeseries we find the time lag of the first peak in their autocorrelation which relates to the firing periodicity. Biologically relevant firing pixels are distinguished from stochastic image noise by having a longer period and were automatically detected as those with peaking time greater than a minimum time threshold. This threshold was determined as the lower threshold computed by applying 3-class Otsu thresholding to all pixel peaking times[1]. All detected firing pixels were assembled in a binary image. Connected component analysis was applied to remove firing regions with area smaller than 25 pixels and greater than mean$\pm$3 standard deviation pixels. The summed area of the remaining firing regions within the tissue area is the firing signal area. The tissue area was automatically determined by thresholding the maximum temporal

projection of the normalized timelapse frames and binary filling all holes. The intensity threshold was computed as the lower threshold after applying 3-class Otsu thresholding on the image intensity.

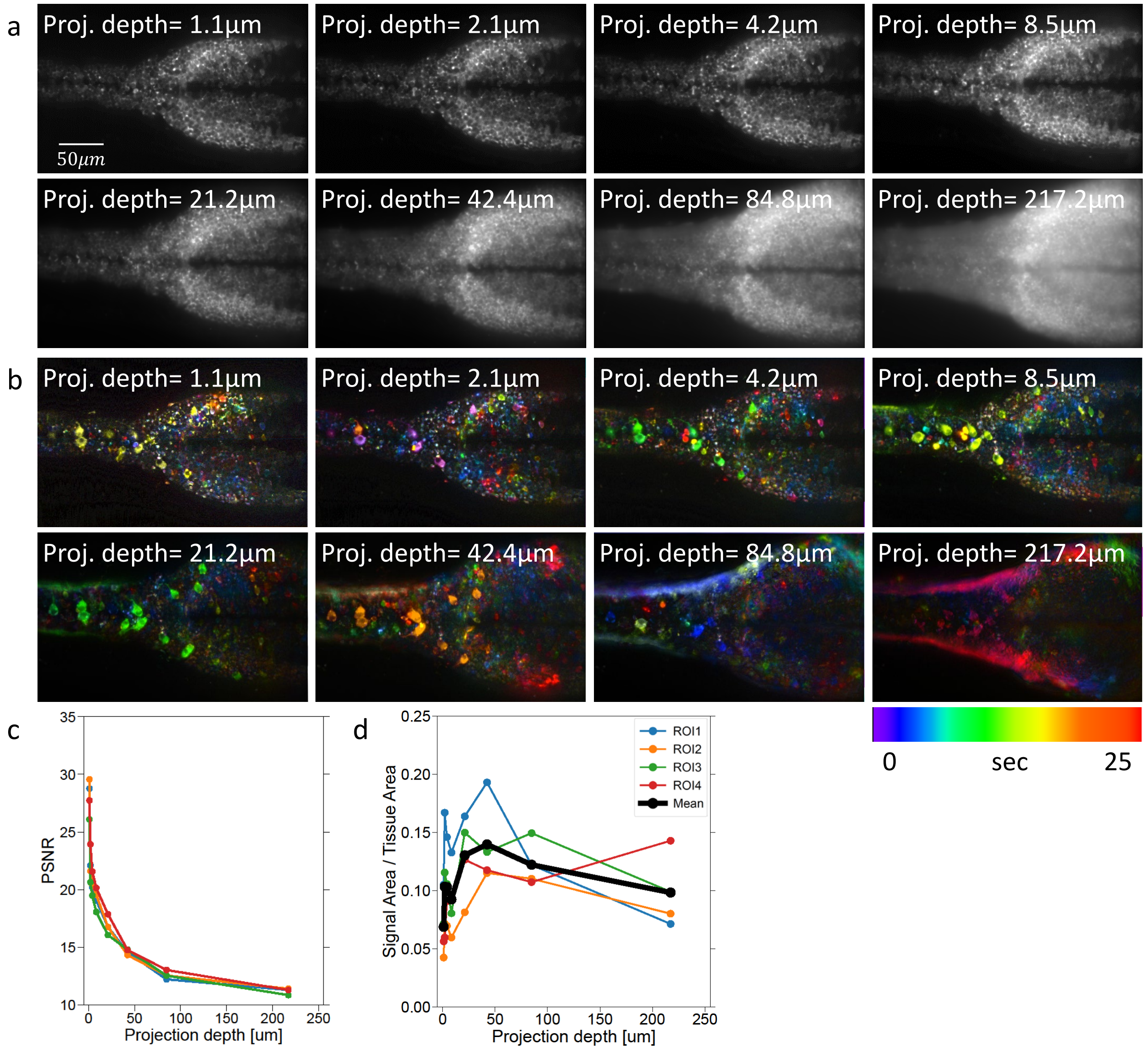

Supplementary Figure 10. Effect of projection depth in calcium imaging. a The average temporal projection of the timelapse at different projection depths. **b** The temporal color-coded maximum intensity projection of the timelapse after the average of the entire time series has been subtracted. **c** Quantification of the image contrast using PSNR. **d** Quantification of the number of pixel firing events at different projection depths. Please note that 4 datasets acquired from the same fish are plotted in C and D, whereas in a and b only one dataset (ROI1) is presented as an example. Source data are provided as a Source Data file.

As a result, this analysis revealed that there exists a projection depth range (~4.2-42.4µm) where more biologically-relevant firing events can be captured without excessive image blurring (**Supplementary Figure 10d**). Off note, this range exceeds the depth of focus of a spinning disk microscope (on the order of 1 micron, or less).

We also applied CaImAn, a popular method to segment single neurons from calcium imaging data[2]. In a single zebrafish case, we could segment 87, 120, 101, 128, 201, 262, 215, and 206 regions of interest (ROIs) from the projection depth of 1.1, 2.1, 4.2, 8.5, 21.2, 42.4, 84.8, and 217.2 µm, respectively (**Supplementary Figure 11a**). However, when we looked closer at the individual ROIs across the different projection depths (**Supplementary Figure 11b**), most ROIs from a thin projection depth (~1.1-21.2 µm) showed round shapes that looked like single neurons, but many ROIs from a thick projection depth (~42.4-217.2 µm) exhibited non-round irregular shapes, frequent overlapping, and increased ROI-size heterogeneity. Particularly in the correlation image of the data with 217.2 µm projection depth, there seemed to be many ROIs that would not be single neurons. This could be a result of projecting too much information (neuros and image blurring) into a single projection.

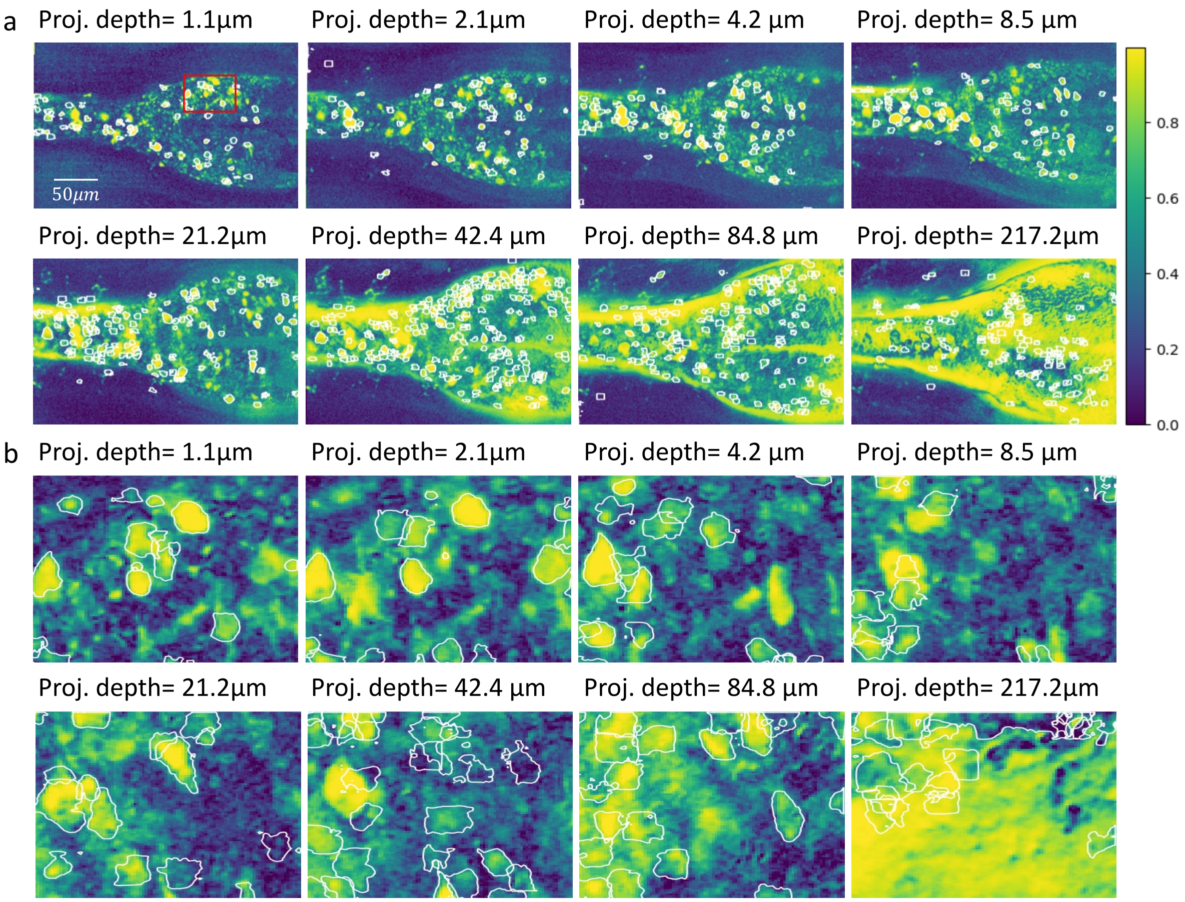

Supplementary Figure 11. Quantification of firing neurons with CaImAn. **a** The correlation image with the annotated ROIs at different projection depths. **b** The zoom-in of the red box in **a**.

In conclusion, props can optimize the projection depth to balance the number of firing events that can be captured and then later detected. Such optimization cannot be done with confocal or spinning disk confocal microscopy because the depth of focus is fixed, and typically too thin (≤1µm) to encompass an entire neuron cell body.

<u>Some details of the</u> CaImAn <u>image analysis:</u>

1. Preprocessing of the data: All datasets have been downsampled 4x (2048x1448 to 512x362), bleach corrected, and then registered with pairwise multiscale affine transformation.
2. CaImAn[2]: The CaImAn pipeline was downloaded from the repository (https://github.com/flatironinstitute/CaImAn). Among the segmentation parameters, the number of background components was set to be two.

Supplementary Note 4: Acquisition speed comparison between 3D stacking and projection imaging.

Here we explore how the acquisition time for a 3D stack compares to a props image, or a series of props images given current camera capabilities. As an example, we compare how fast a 3D stack can be acquired for the Drosophila embryo imaging shown in **Figure 3a**, and how fast props can be acquired.

The calculation is based on the instruction manual of ORCA-Flash4.0 V3 Digital CMOS camera (C13440-20CU). We use the normal area mode of the camera to acquire the 3D stack and light-sheet readout mode to get the props image, and the operation mode of the camera is external trigger mode (edge trigger) for both.

The raw props images shown in **Figure 3a** have 2048x2048 pixels and the frame rate is limited to a maximum of 49 fps when using a rolling shutter width of 4 pixels. When the rolling shutter of the camera is set to 80 pixels, the maximum frame rate drops to 48 Hz in theory (per Hamamatsu's manual). In practice, we acquired the images with a frame rate of 47.8 fps.

Using the full chip, the corresponding FOV after compression is 307.2um x 217.2 um in the top-down view and the imaging depth is around 8.5 um for the chosen rolling shutter width. To acquire the same volume as a 3D stack, we need to capture 1034 planes (217.2 µm/0.21 µm, scan range/step size) and each plane should have an image size of 2048x40 pixels. The frame rate for images with 80 vertical lines is 670 fps, so the volume rate is ~0.65 Hz (670 fps/1034 frames), which is around 74 times slower than using props.

In the extreme case, we can compare the speed between 3D stacking with the narrowest width (4 pixel) this camera can acquire. The frame rate of the 2048x4 subarray in external trigger mode is 892 Hz, which increases the 3D stacking speed to 0.86 Hz. The fastest frame rate we can get is 25655 Hz in free running mode. It can increase the overall imaging speed to 24.8 Hz but will bring a challenge in software control and data streaming.

We also note that the imaging speed for sub-array setting has been greatly improved in the newly developed cameras. As an example, the frame rate of the ORCA-Fusion for a 2304x4-pixel image is 18600 Hz, which increased the 3D stacking speed to 16.1 Hz. However, the imaging speed for the full chip is also increased to 88.8 Hz, thus the props imaging mode would still be 5.5 times faster.

As to the data size, a 2048x2048-pixel 16-bit props image takes 8.4 MB storage space, while a 2048x40x1034-pixel volume will need 169.4 MB, which is 20 times more. Therefore, for long term times lapse imaging of biological processes, props eases data storage requirements.

Acquiring multiple, axially shifted props increases sample exposure, as the light-sheet is scanned through the sample each time. Thus, a projection stack is only recommended when the speed advantage over conventional z-stack acquisition is needed.

Supplementary Note 5: Procedure to calibrate the control signals.

Here we detail how we calibrate the scan parameters, in the example of an OPM system. We used a micrometer (2280-16, Ted Pella) to calibrate the magnification of the detection system, as well as the scanning galvo of the OPM (i.e., the galvo mirror that scans the light-sheet and de-scans the fluorescence). These are necessary steps even for a conventional OPM (i.e., to know its pixel size and scanning step size for stack acquisitions).

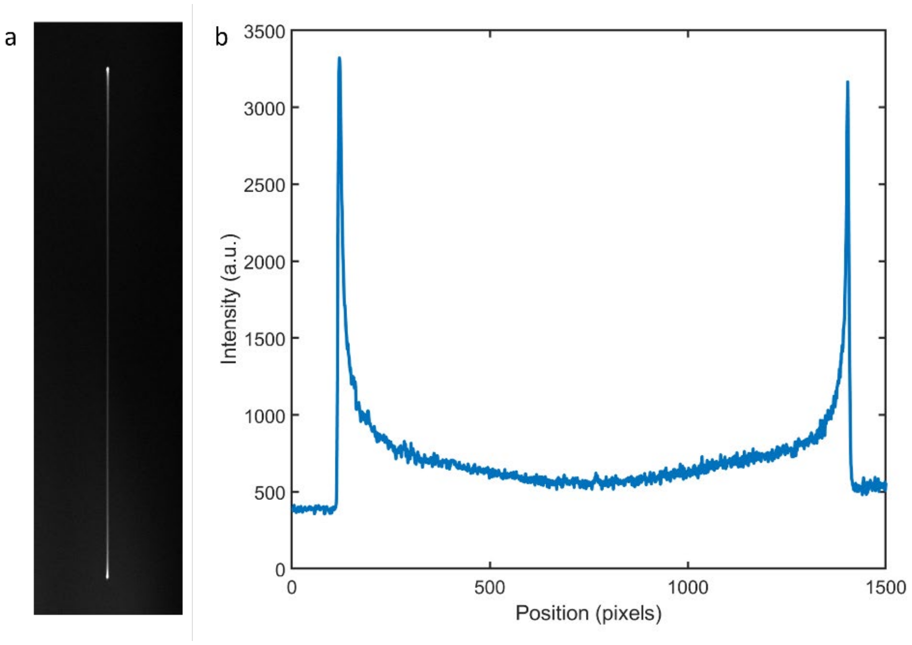

Supplementary Figure 14 Shear galvo calibration. a the image of a scanning laser spot on the camera, which is dynamically scanned with the shear galvo. **b** the profile along the line in a. Source data are provided as a Source Data file.

To calibrate the shear galvo, we used an alignment laser, which propagates along the detection path of the OPM (e.g. illuminate from the space between the objective and the tube lens to the camera). We first adjusted the laser spot to the center of the camera, then added an input sawtooth signal to the shear galvo and measured the scanning distance of the laser spot on the camera. As an example, with a 1V peak-to-peak control signal, we acquired an image of the laser spot scanned by the shearing a galvo, shown as **Supplementary Figure 14a**. We measured the length of the line as 1284 pixels (**Supplementary Figure 14b**), then we obtained the relationship of the shear galvo control signal and shear distance as 7.79e-4 V/pixel.

For the projection imaging shown within this manuscript, the shearing galvo signal is proportional to the scanning galvo's control signal. We can calculate the ratio between them, multiply it to the scanning galvo's control signal to control the shearing galvo. In our case, we use a scaling amplifier (SIM983, Standard Research Systems), so that we can avoid synchronization issue between the two signals. For example, when the oblique imaging plane is tiled at 45 degrees, for 100-µm scanning range, the shearing range will be 70.7 µm to form the side view. 70.7 µm corresponds to 471.4 pixels with 0.15 µm pixel size, and we adjust the region of interest on the camera accordingly.

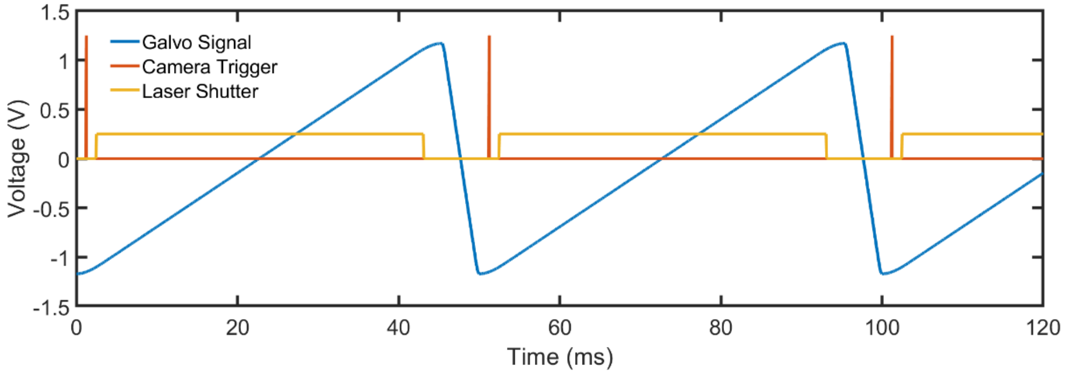

Supplementary Figure 15. Example control signals for props. The blue galvo signal drives the OPM scanning galvo, and a scaled version of the signal is applied to the shearing galvo. Source data are provided as a Source Data file.

In **Supplementary Figure 15**, an example of the waveforms is shown. The blue galvo signal is applied to the scanning galvo of the OPM. A scaled version of the blue signal is applied to the shearing galvo. The camera trigger starts when the galvo signal enters the linear range.

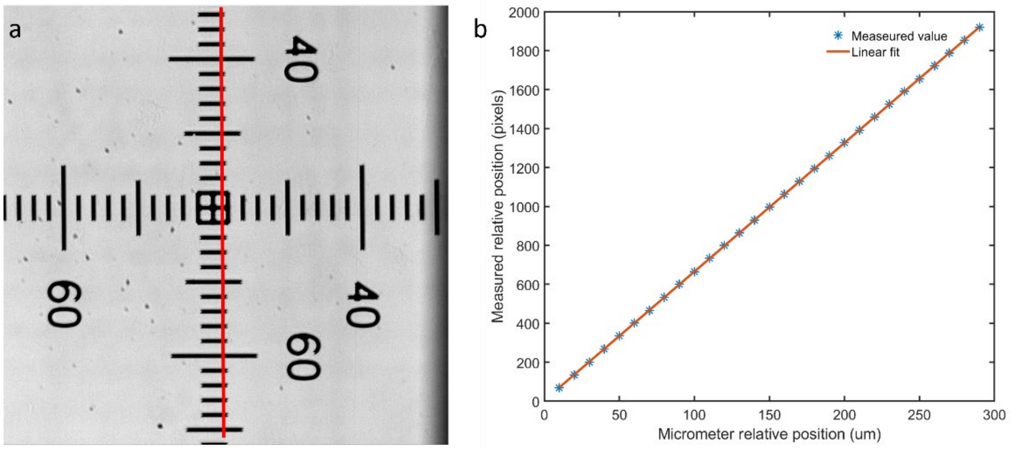

Supplementary Figure 16. Assessing the linearity of a projection image. a Microruler as imaged with OPMprops**.** The shear direction runs vertically. **b** Comparison of micrometer increments to projection pixel distances along the red curve in **a**. Source data are provided as a Source Data file.

Even though our OPM system we used in this work used a single galvo, as detailed in previous work, the linearity of the resulting projection imaging was sufficiently high: in **Supplementary Figure 16a**, a projection image of a micro-ruler is shown, where the vertical dimension is the shear direction. As shown in **Supplementary Figure 16b**, the projected increments of the micro-ruler follow a linear curve.

Please note, given that this is an OPM with a tilted image plane, a stationary image of the ruler would not be sharp in the vertical direction. Only in a projection image, does the ruler becomes visible in both directions.

For the work presented herein, our microscope software did not automatically calculate the necessary scan ranges for a given camera ROI. Instead, we manually calculated the values and applied them for each image acquisition.

Supplementary Table

Figure	microscope	Exposure time (ms)	Acquisition rate (Hz)	Pixel size (µm)	Field of view (µm x µm)	Projection depth (µm)	Laser power (mW)
1h	Field Synthesis	200	5	0.104	106.5x69.9	2.1, 6.2, 14.6, and GS	NA
1i-j	Meso OPM	100	Single exposure	1.15	2355x805	(i): 8<br>(j): 325	6
2a-b	OPM	50	20	0.15	307.2x217.2	(a): GS<br>(b): 4.2	1.64
2c-d	OPM	(a): 50<br>(b): 16.7	(c): 20<br>(d): 19.6	0.15	307.2x108.6	(c): GS<br>(d): 4.2	2.00
2e	OPSIM	60	1.82	0.114 (raw)<br>0.057 (processed)	64x64	2.4	0.7
2h-i	OPM	95	10	0.15	(h): 307.2x132.0<br>(i): 307.2x203.2	2.1	2.00
3a	OPM	20	47.8	0.15	307.2x217.2	8.5	1.62
3e-f	OPM	50	(e): 20<br>(f): 19.8	0.15	307.2x217.2	(f): 4.2	1.78
3i-k	OPM	50	10	0.15	307.2x217.2	4.2	1.46
Suppl 1	Field Synthesis	25	NA, single timepoint	0.104	106.5x140.3	none	0.08
Suppl 2	OPM	50	19.8	0.15	307.2x217.2	shown in the figure	2.00
Suppl 3	Field Synthesis	200	NA, single timepoint	0.104	80.7x106.5	4.2	NA
Suppl 4	OPSIM	60	1.82	0.114	64x64	2.4	0.7
Suppl 5	OPM	20	50, frame	0.15	108.6x408.6	NA	0.31
Suppl 9	SoRa	(a): 100<br>(b): 200	NA, single timepoint	0.1625	(a) 83.2x83.2<br>(b): 374.4x374.4	NA	NA

**Suppl 10**	**OPM**	**100**	**10**	**0.15**	**307.2x217.2**	**shown in the figure**	**1.76**
**Suppl 11**	**OPM**	**100**	**10**	**0.15**	**307.2x217.2**	**shown in the figure**	**1.76**
**Suppl 12**	**Field Synthesis: (a,c)**<br>**SoRa: (b,d)**	**(a,c): 200**<br>**(b,d): 100**	**NA, single timepoint**	**(a,c): 0.104**<br>**(b,d): 0.1625**	**83.2x60.7**	**(a,c): 0.52**	**NA**
**Suppl 13**	**OPM**	**(a): 926**<br>**(b,c): 15**	**(a): 0.097**<br>**(b,c): 0.067**	**(a): 0.15**<br>**(b,c): 0.15**	**(a): 307.2x217.2**<br>**(b,c): 307.2x243**	**(a): 4.2**	**(a): 0.47**<br>**(b,c): 0.31**
**Suppl Movie 1**	**Meso OPM**	**100**	**NA, single timepoint**	**1.15**	**2355x805**	**4-362**	**6**
**Suppl Movie 2**	**Meso OPM**	**200**	**NA, single timepoint**	**1.15**	**1024x600**	**4-487**	**10**
**Suppl Movie 3**	**OPM**	**50**	**19.8**	**0.15**	**307.2x217.2**	**shown in the figure**	**2.00**
**Suppl Movie 4**	**OPM**	**95**	**10**	**0.15**	**307.2x172.7**	**2.1**	**2.00**
**Suppl Movie 5**	**OPM**	**20**	**47.8**	**0.15**	**307.2x217.2**	**8.5**	**1.62**
**Suppl Movie 6**	**OPM**	**50**	**(left): 20**<br>**(right): 19.8**	**0.15**	**307.2x217.2**	**(right): 4.2**	**1.62**
**Suppl Movie 7**	**OPM**	**16.6**	**1.99**	**0.15**	**307.2x217.2**	**4.2**	**1.78**
**Suppl Movie 8**	**OPM**	**50**	**10**	**0.15**	**307.2x217.2**	**4.2**	**1.46**
**Suppl Movie 9**	**Field Synthesis**	**(left): 25**<br>**(right): 200**	**NA, single timepoint**	**0.104**	**83.2x60.7**	**(right): 0.52**	**NA**
**Suppl Movie 10**	**OPM**	**926**	**0.097**	**0.15**	**307.2x217.2**	**4.2**	**0.47**

Supplementary Table 1 – Image acquisition parameters

Supplementary References

1. N. Otsu, "A threshold selection method from gray-level histograms," IEEE transactions on systems, man, and cybernetics **9**, 62-66 (1979).
2. A. Giovannucci, J. Friedrich, P. Gunn, J. Kalfon, B. L. Brown, S. A. Koay, J. Taxidis, F. Najafi, J. L. Gauthier, P. Zhou, B. S. Khakh, D. W. Tank, D. B. Chklovskii, and E. A. Pnevmatikakis, "CaImAn an open source tool for scalable calcium imaging data analysis," eLife **8**, e38173 (2019).
