# Supplementary material for: Projective light-sheet microscopy with flexible parameter selection
Source: Nat Commun. 2024 Mar 29;15:2755. doi: 10.1038/s41467-024-46693-y (PMC10980818; doi:10.1038/s41467-024-46693-y)
Supplement: Supplementary file 2 — Reporting Summary [file 41467_2024_46693_MOESM2_ESM.pdf]

nature portfolio

Corresponding author(s): Reto Fiolka

Last updated by author(s): Feb 12, 2024

Reporting Summary

Nature Portfolio wishes to improve the reproducibility of the work that we publish. This form provides structure for consistency and transparency in reporting. For further information on Nature Portfolio policies, see our Editorial Policies and the Editorial Policy Checklist.

Statistics

For all statistical analyses, confirm that the following items are present in the figure legend, table legend, main text, or Methods section.

n/a	Confirmed	
[ ]	[x]	The exact sample size (*n*) for each experimental group/condition, given as a discrete number and unit of measurement
[x]	[ ]	A statement on whether measurements were taken from distinct samples or whether the same sample was measured repeatedly
[x]	[ ]	The statistical test(s) used AND whether they are one- or two-sided<br>*Only common tests should be described solely by name; describe more complex techniques in the Methods section.*
[x]	[ ]	A description of all covariates tested
[x]	[ ]	A description of any assumptions or corrections, such as tests of normality and adjustment for multiple comparisons
[x]	[ ]	A full description of the statistical parameters including central tendency (e.g. means) or other basic estimates (e.g. regression coefficient) AND variation (e.g. standard deviation) or associated estimates of uncertainty (e.g. confidence intervals)
[x]	[ ]	For null hypothesis testing, the test statistic (e.g. *F*, *t*, *r*) with confidence intervals, effect sizes, degrees of freedom and *P* value noted<br>*Give P values as exact values whenever suitable.*
[x]	[ ]	For Bayesian analysis, information on the choice of priors and Markov chain Monte Carlo settings
[x]	[ ]	For hierarchical and complex designs, identification of the appropriate level for tests and full reporting of outcomes
[x]	[ ]	Estimates of effect sizes (e.g. Cohen's *d*, Pearson's *r*), indicating how they were calculated

Our web collection on statistics for biologists contains articles on many of the points above.

Software and code

Policy information about availability of computer code

Data collection	The LabView -based control software used within this manuscript to control the OPM and LLSM systems is freely available for nonprofit institutions through a Material Transfer Agreement with UT Southwestern Medical Center.
Data analysis	Matlab, Fiji (https://fiji.sc/), CalmAn (https://github.com/flatironinstitute/CalmAn) . Code used for our analysis is deposited in a public github repository: https://github.com/AdvancedImagingUTSW/manuscripts/tree/main/2024-chen-PROPS

For manuscripts utilizing custom algorithms or software that are central to the research but not yet described in published literature, software must be made available to editors and reviewers. We strongly encourage code deposition in a community repository (e.g. GitHub). See the Nature Portfolio guidelines for submitting code & software for further information.

Data

Policy information about availability of data

All manuscripts must include a data availability statement. This statement should provide the following information, where applicable:

- Accession codes, unique identifiers, or web links for publicly available datasets
- A description of any restrictions on data availability
- For clinical datasets or third party data, please ensure that the statement adheres to our policy

We have no restrictions on data availability. Figure and movie data is available at https://zenodo.org/records/10 182659

Research involving human participants, their data, or biological material

Policy information about studies with human participants or human data. See also policy information about sex, gender (identity/presentation), and sexual orientation and race, ethnicity and racism.

Reporting on sex and gender	Not applicable
Reporting on race, ethnicity, or other socially relevant groupings	Not applicable
Population characteristics	Not applicable
Recruitment	Not applicable
Ethics oversight	Not applicable

Note that full information on the approval of the study protocol must also be provided in the manuscript.

Field-specific reporting

Please select the one below that is the best fit for your research. If you are not sure, read the appropriate sections before making your selection.

☒ Life sciences ☐ Behavioural & social sciences ☐ Ecological, evolutionary & environmental sciences

For a reference copy of the document with all sections, see nature.com/documents/nr-reporting-summary-flat.pdf

Life sciences study design

All studies must disclose on these points even when the disclosure is negative.

Sample size	We conducted each imaging experiment a minimum of 3 times to demonstrate the capability and replicability of our method. Our sample size was selected based on the established standards in the field and was sufficient to support our conclusions.
Data exclusions	No data was excluded.
Replication	For each experiment we made sure to perform at least n=3 repeats to ensure technical replicability.
Randomization	Randomization was not applicable because there was no group allocation in this study.
Blinding	Blinding was not applicable because there was no group allocation in this study.

Reporting for specific materials, systems and methods

We require information from authors about some types of materials, experimental systems and methods used in many studies. Here, indicate whether each material, system or method listed is relevant to your study. If you are not sure if a list item applies to your research, read the appropriate section before selecting a response.

Materials & experimental systems

n/a	Involved in the study	
☒	☐	Antibodies
☐	☒	Eukaryotic cell lines
☒	☐	Palaeontology and archaeology
☐	☒	Animals and other organisms
☒	☐	Clinical data
☒	☐	Dual use research of concern
☒	☐	Plants

Methods

n/a	Involved in the study	
☒	☐	ChIP-seq
☒	☐	Flow cytometry
☒	☐	MRI-based neuroimaging

Eukaryotic cell lines

Policy information about cell lines and Sex and Gender in Research

Cell line source(s)	The U-2 OS cells were a gift from Jodi Nunnari, UC Davis. The A375 cells were obtained from ATCC(CRL-1619).
Authentication	The A375 and U-2 OS cells were not authenticated.
Mycoplasma contamination	The A375 and U-2 OS cells were tested negative for mycoplasma contamination.
Commonly misidentified lines (See ICLAC register)	No misidentified cell lines were used in this study.

Animals and other research organisms

Policy information about studies involving animals; ARRIVE guidelines recommended for reporting animal research, and Sex and Gender in Research

Laboratory animals	Danio Rerio (Zebrafish), strains: Tg(kdrl:Hsa.HRAS-mcherry), Tg(elavl3:soma-GCaMP7f) and Tg(kdrl:EGFP) in a casper background. Age: 3-5 dpf, therefore the sex of the organism was not yet determined.<br><br>Drosophila melanogaster (fruitfly): Strainl: transgenic files expressing UAS-ActinSC-RFP or UAST-jGCaMP7s-CAAX, bred with transgenic lines expressing Gal4 in mesoderm (Mef2-Gal4). Age: up to 20 hours. Strain2: genotype sqh>Gap43::mCherry. Strain3: genotype sqhFRB-GFP; +; gap43::mCherry/TM3, Sb. Age: up to 3 hours. The sex of the organisms was not determined.
Wild animals	No wild animals were used.
Reporting on sex	The imaging experiments were conducted using both male and female animals, and the imaging results were independent of gender.
Field-collected samples	No field-collected samples were used.
Ethics oversight	Animal procedures and protocols were approved by the University of Texas Southwestern Medical Center. Zebrafish work described in this manuscript has been approved and conducted under the oversight of the Institutional Animal Care and Use Committee (IACUC) at UT Southwestern under APN 2016-101805 to Gaudenz Danuser.

Note that full information on the approval of the study protocol must also be provided in the manuscript.

Plants

Seed stocks	Not applicable
Novel plant genotypes	Not applicable
Authentication	Not applicable
