# Supplementary material for: Projective light-sheet microscopy with flexible parameter selection
Source: Nat Commun. 2024 Mar 29;15:2755. doi: 10.1038/s41467-024-46693-y (PMC10980818; doi:10.1038/s41467-024-46693-y)
Supplement: Supplementary file 3 — Description of Additional Supplementary Files [file 41467_2024_46693_MOESM3_ESM.pdf]

Supplementary Movies

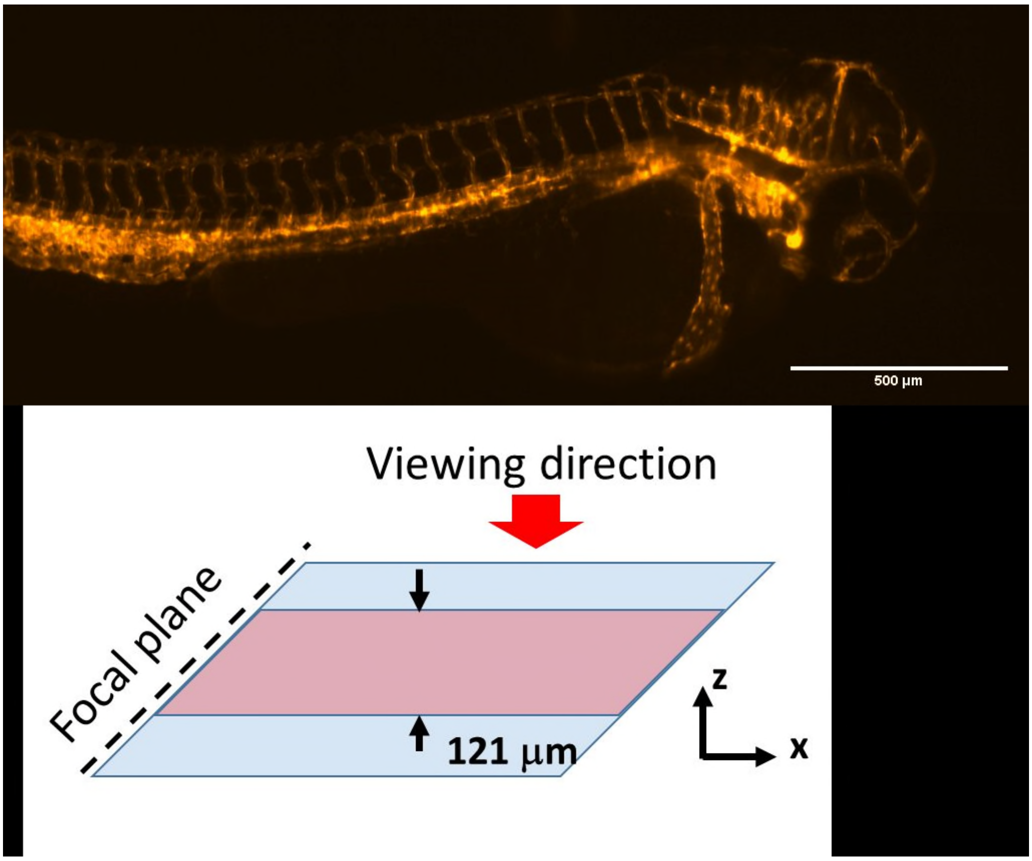

Supplementary Movie 1 Variation of projection depth. Zebrafish embryo, labeled with Tg(kdrl:EGFP), as imaged with mesoscopic OPMprops under different projections depth. The cartoon below illustrates what portion (red shading) of the overall volume spanned by the focal sweep (blue shading) is being projected.

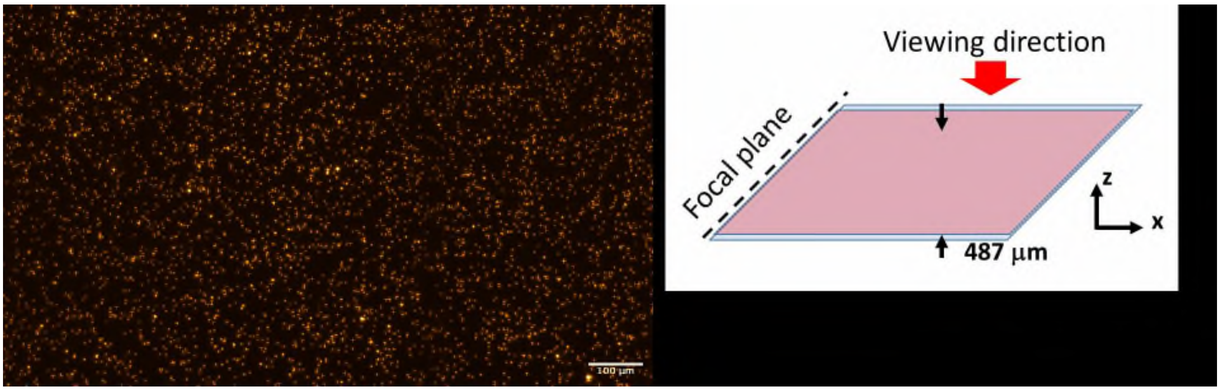

Supplementary Movie 2 Variation of projection depth using beads in Agarose gel. 500nm fluorescent nanospheres, embedded in an Agarose gel, as imaged with mesoscopic OPMprops. The cartoon on the right illustrates what portion (red shading) of the overall volume spanned by the focal sweep (blue shading) is being projected.

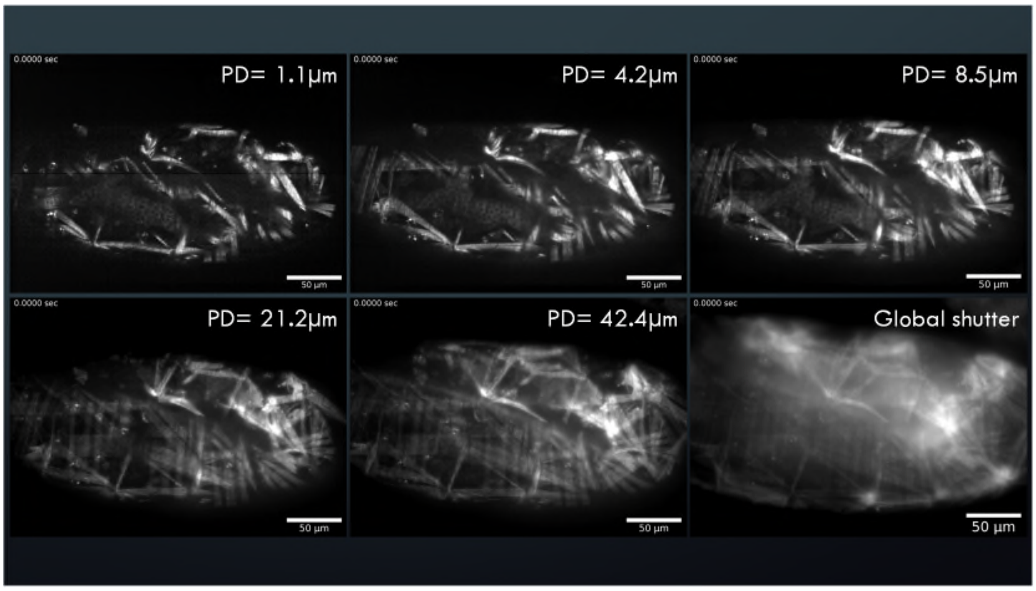

Supplementary Movie 3 Variation of projection depth. Drosophila embryo, labeled with actin5C-RFP, as imaged with OPMprops with different rolling shutter widths (resulting projection depth, PD, indicated in microns). Bottom right image shows a projection using global shutter. The imaging speed is 19.8Hz.

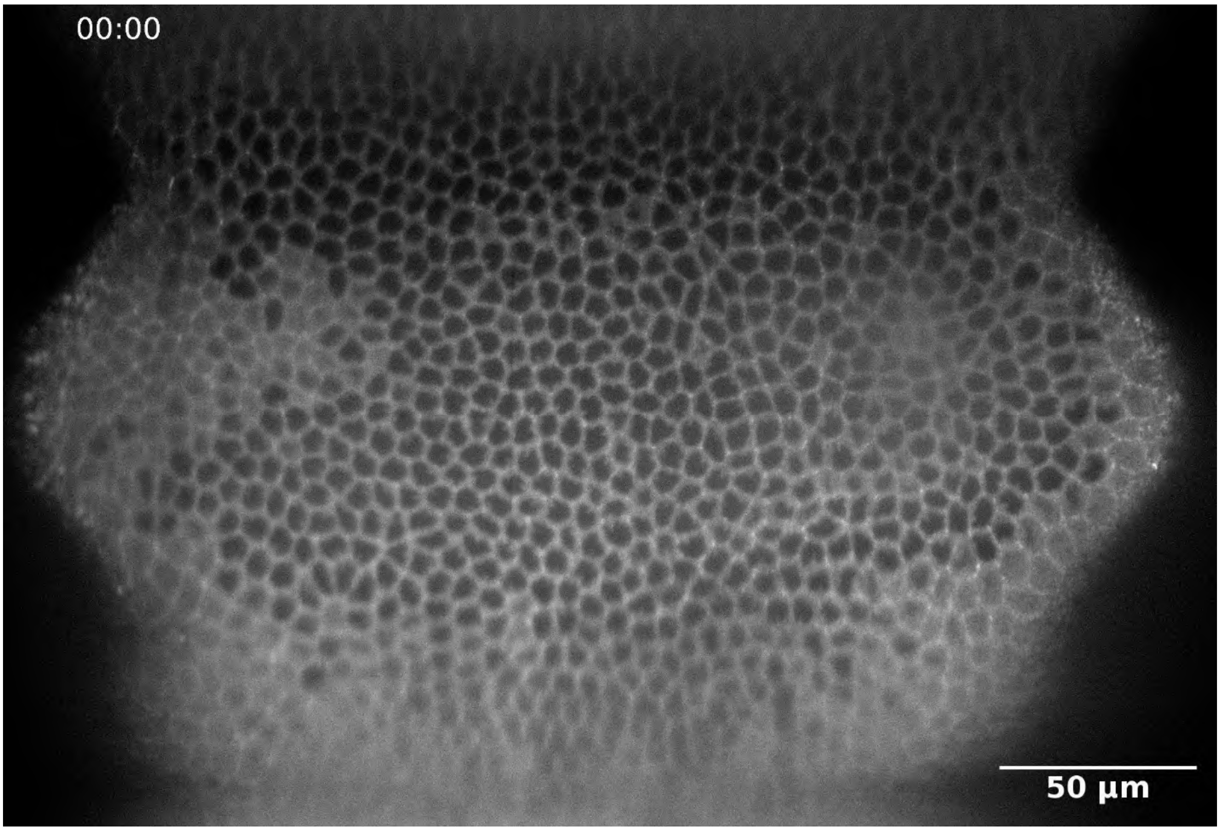

Supplementary Movie 4 Curved projections of a Drosophila embryo. Curved projection imaging of ventral furrow formation in a Drosophila embryo labeled with myosin-GFP. The curved surface is described in Supplementary Note 2. The projection depth is 2 µm. The images that make up of the movie were taken every 10 seconds with the exposure time of 95 ms.

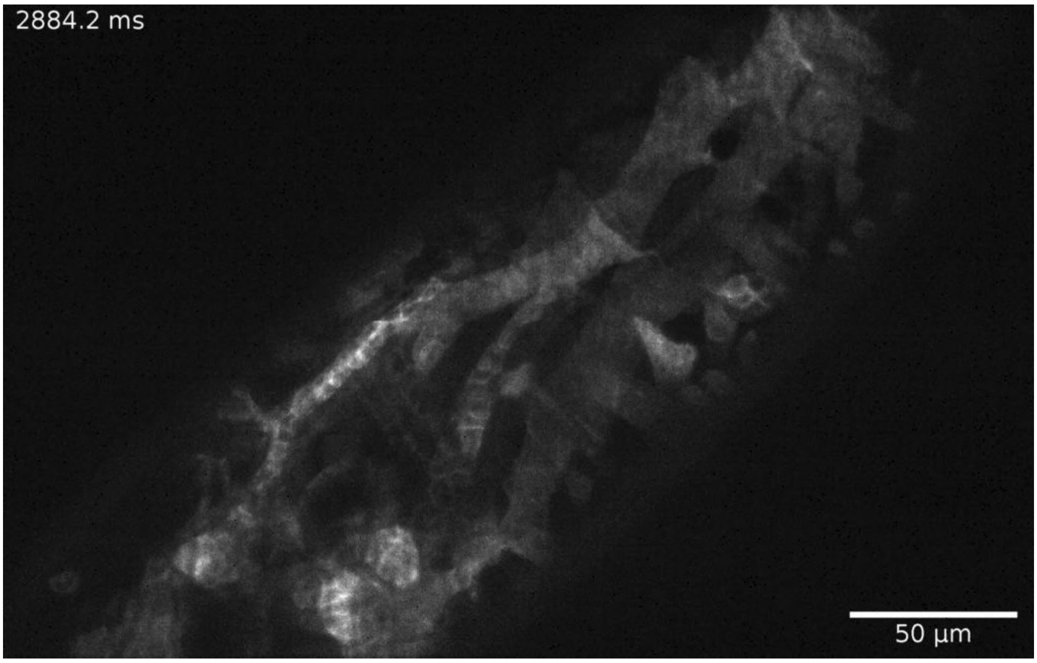

Supplementary Movie 5 Calcium dynamics in a Drosophila embryo imaged with OPMprops. A *Drosophila* embryo expressing jGCaMP7s-CAAX imaged at 50Hz rate. The projection depth is 8.5µm.

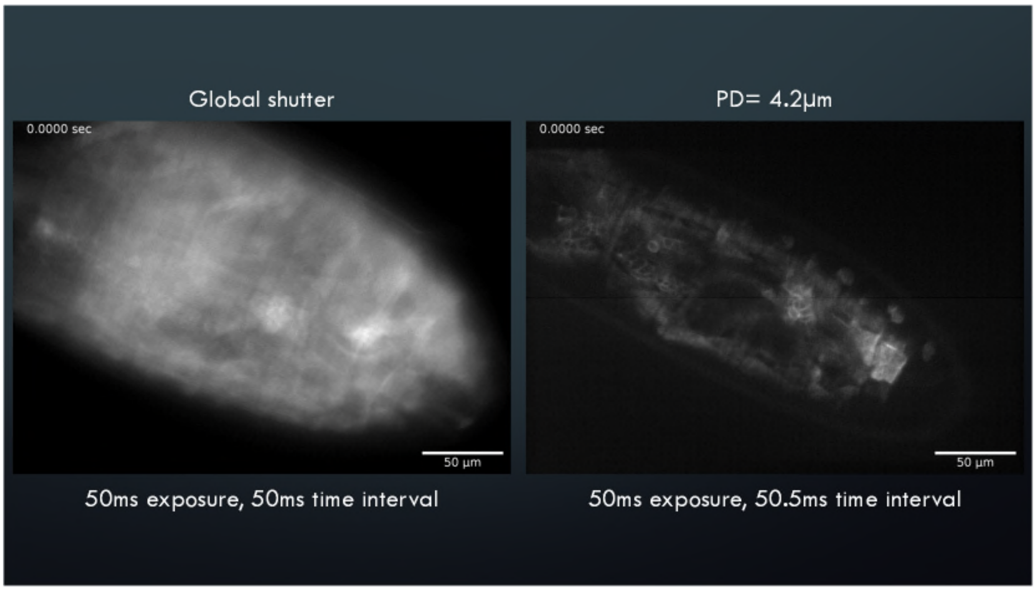

Supplementary Movie 6 Calcium dynamics in a Drosophila embryo as imaged with OPMprops.
Projection imaging (global shutter) and props imaging (PD= 4.2µm) of a *Drosophila* embryo expressing jGCaMP7s-CAAX at 20Hz rate.

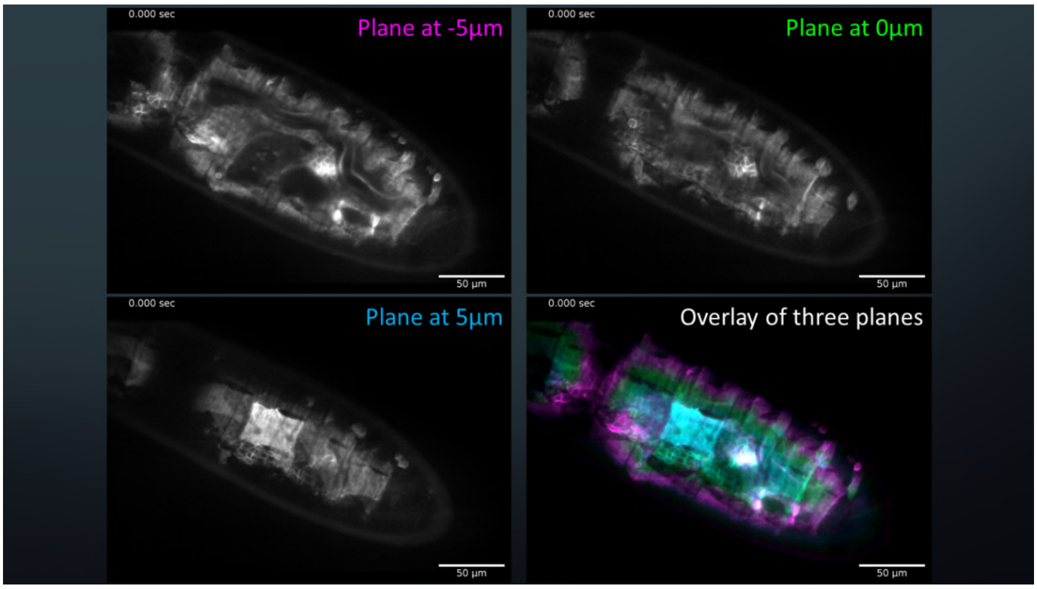

Supplementary Movie 7 Projection imaging at different axial depth. Three projections at different axial positions using OPMprops, and the overlay of three projections of a *Drosophila* embryos expressing jGCaMP7s-CAAX. The axial separation between each projection is 5 microns. The projection depth is 4.2µm. The imaging speed is 2Hz.

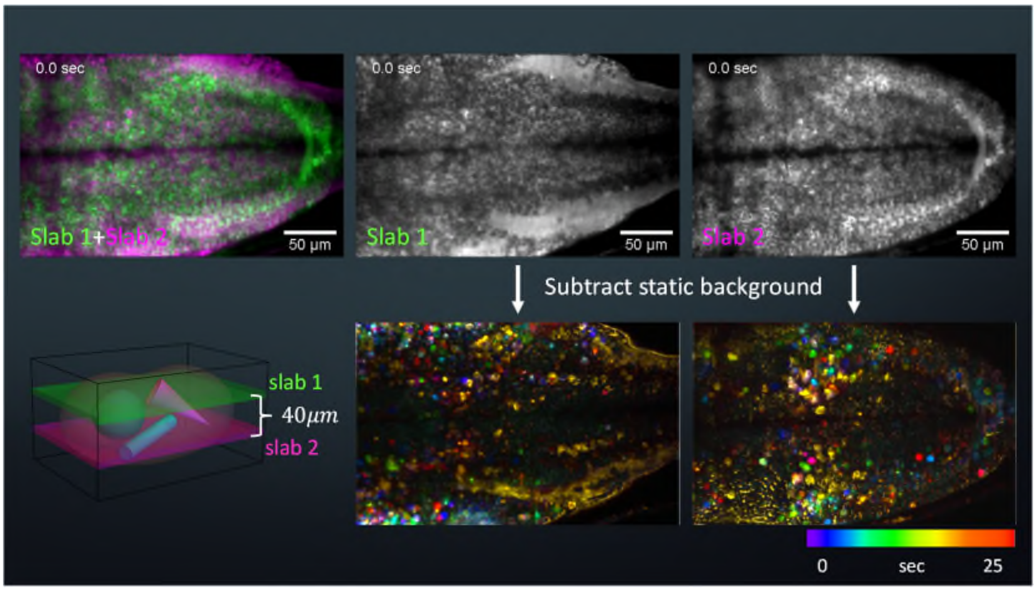

Supplementary Movie 8 Projection imaging of Calcium firing in zebrafish brain at different axial depth (related to figure3 i-n). Two projections acquired with OPMprops at axial positions separated by 40 µm. The projection depth of both slabs is 4.2µm. The imaging speed is 10Hz. Zebrafish brain was labeled with Tg(elavl3:soma-GCaMP7f) targeting the cell body.

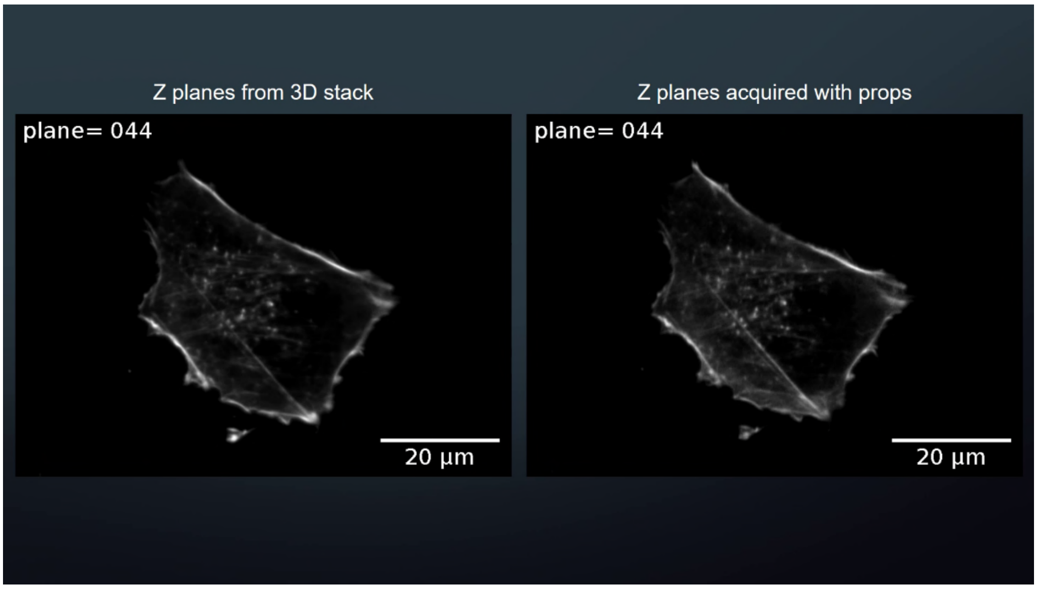

Supplementary Movie 9 Comparison of a conventionally acquired 3D stack, and one assembled from a series of projections. Fixed A375 cells labeled with F-tractin-EGFP acquired with a Field Synthesis light-sheet microscope. Left: z planes from a conventionally acquired 3D stack, which was computationally de-skewed and rotated. Right: z planes acquired directly with OPMprops.

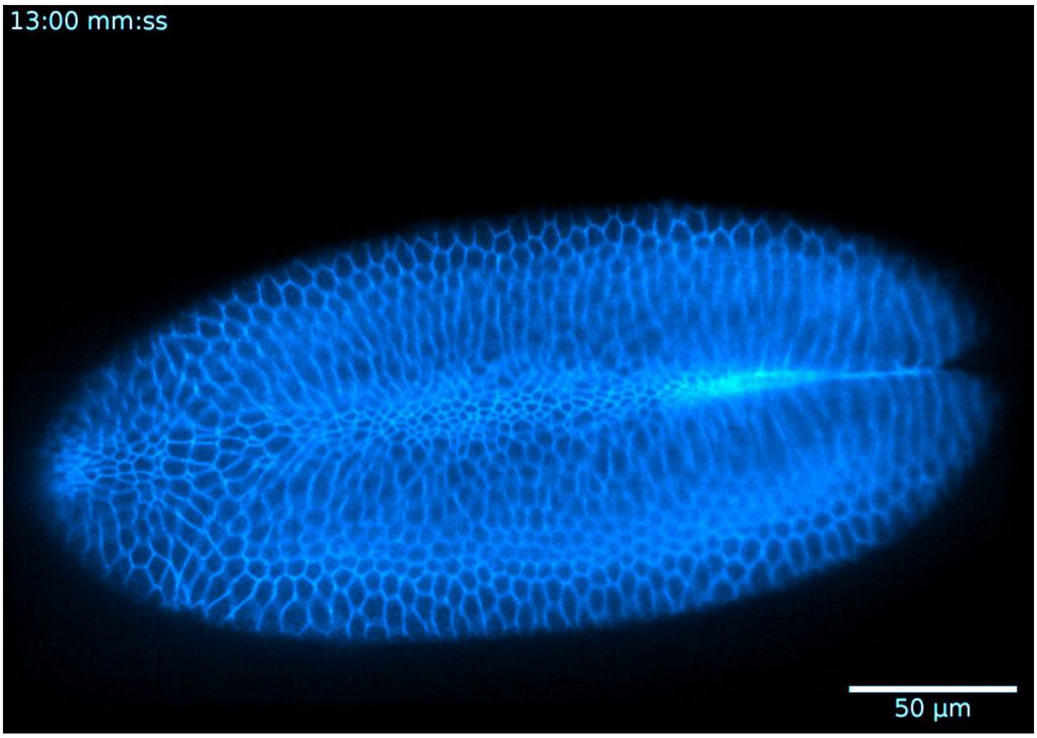

Supplementary Movie 10 ventral furrow formation in a Drosophila embryo as imaged with OPMprops. Projection imaging of ventral furrow formation in a Drosophila embryo labeled with GAP43 mCherry. The projection depth is 4.2µm.
